# Supplementary material for: Inferring pattern-driving intercellular flows from single-cell and spatial transcriptomics
Source: Nat Methods. 2024 Aug 26;21(10):1806–17. doi: 10.1038/s41592-024-02380-w (PMC11466815; doi:10.1038/s41592-024-02380-w)
Supplement: Supplementary file 1 — Supplementary Notes, Supplementary Results, Supplementary Tables 1–4 and Supplementary Figures 1–11. [file 41592_2024_2380_MOESM1_ESM.pdf]

nature methods

Supplementary information https://doi.org/10.1038/s41592-024-02380-w

Inferring pattern-driving intercellular flows from single-cell and spatial transcriptomics

In the format provided by the authors and unedited

Table of Contents

Supplementary Notes 2
Supplementary Results 7
References 16
Supplementary Figures 23

Supplementary Notes

Example FlowSig walkthrough

For additional clarity, we briefly demonstrate how FlowSig is applied to the stimulated pancreatic islets dataset generated by Burkhardt et al.[1]. FlowSig analysis of the pancreatic islets dataset can be broken down into the following steps:

1. We generate cell type annotations for the pancreatic islets data. We first normalized the gene expression counts such that each cell had $10,000$ total counts and then log-transformed the normalized data. We integrated the control (Ctrl) and perturbed (IFN-γ) datasets using *Scanorama*[2], which generated an integrated low-dimensional latent embedding. We then calculated the k-Nearest Neighbors graph using this embedding (seting $k = 30$). Leiden clustering was performed on the neighbor graph, setting $resolution = 0.2$, which generated five clusters. Using cell type markers from the literature[3], we annotated the clusters as Alpha, Beta 1, Beta 2, Beta 3, and Delta cells.
2. Using CellChat, we use the log-transformed data and cell type labels inferred from the previous step as input. We applied CellChat to the Control and IFN-γ datasets separately. For each condition-specific data , we used CellChat's *rankNet* function to obtain the list of all significant ligand-receptor interactions. We focus only on interactions involving secreted, diffusible ligands.
3. FlowSig uses pyLIGER to calculate the GEMs from the unnormalized gene expression counts, which accounts for both condition-shared and condition-specific contributions when constructing the GEMs. We set the number of GEMs to be 10.
4. We load the ligand-receptor interaction dataframes generated by CellChat and store them as unstructured data, i.e., *adata.uns['cellchat_output']* for the AnnData format, which contains the *'Ctrl'* and *'IFNg'* keys and the corresponding ligand-receptor interaction lists.
5. FlowSig then constructs the "flow expression" matrix.

6. FlowSig performs differential expression analysis on the sets of signal inflow and signal outflow variables separately to infer which signal inflow variables and signal outflow variables are differentially expressed between perturbation condition.
7. FlowSig subsets the original flow expression matrix to only include differentially "flowing" signal inflow and signal outflow variables. All GEM modules are retained.
8. FlowSig uses UT-IGSP to infer the intercellular flow network in the form of a bootstrapped-averaged CPDAG, which includes both directed and undirected edges and their corresponding bootstrap frequencies. We note that UT-IGSP requires us to specify the condition label that we use to split the data, and to specify which condition is the control dataset, so that it can perform conditional invariance testing correctly.
9. We then reorient the undirected edges of the bootstrapped CPDAG such that edges represent biologically consistent information flow. That is, we reorient edges so that they connect signal inflow to GEM nodes, and GEM nodes to other GEM nodes or signal outflow nodes. We also remove any undirected edges that are not oriented in this manner.
10. Finally, we remove low-confidence edges, which we define as edges with bootstrapped edge frequencies below a specified threshold. For the pancreatic islets data, we set the edge frequency threshold to be 0.7.

Generating synthetic data from model simulations

We benchmark FlowSig using synthetic data generated from simulations of various mathematical models describing various intercellular flow situations in biology. Each model that we consider has been validated in previous experimental studies.

For each scenario, we model diffusion across a 2D grid of cells representing the network of nodes. We consider a simple 2D grid of cells, such that interior cells have four neighbors (left, right, up, and down), non-corner border cells have three neighbors, and corner cells have two neighbors. We solve all intercellular flow equations using the Python package *numbalsoda* (https://github.com/Nicholaswogan/numbalsoda), simulating dynamics long

enough such that a steady state has been reached. We use the final timepoint numerical solutions as our synthetic gene expression data.

For all intercellular flow models, we generated five "control" samples and five "perturbed" samples. For each model, we bootstrap aggregated FlowSig results over 500 realizations, calculating the TPR and TNR each time against the known ground truth CPDAG.

All intercellular flow activation mechanisms were derived from mass action kinetics. To this end, for more convenient notation, we define the hill function, $\mathrm{H}(x)$, as:

$$H(x) = \frac{x}{1+x}.$$

Simple inflow-outflow

Here, we model unidirectional intercellular flow induced by spatially diffusing SHH ligand. Inflow due to bound SHH complex formation triggers downstream activation of FOXF1, which then activates outflow of BMP4, which can also diffuse. This model is derived from experimental evidence of this unidirectional flow from epithelial and mesenchymal cells during ureter development, as reported by Bohnenpoll et al.[4]

We model the diffusion and binding of SHH across a static network of cells. While we model diffusion of both SHH and BMP4, as we are only considering unidirectional flow, we need only model the binding of SHH ligand to its receptors. The equations describing the dynamics of SHH ligand, SHH receptor, bound SHH complex, FOXF1, and BMP4 are:

$$\frac{d\left[L_i^{(\mathrm{SHH})}\right]}{dt} = \mathcal{D}^{(\mathrm{SHH})} \sum_{j=1}^{N} \Delta_{ij} \left[L_i^{(\mathrm{SHH})}\right] - \alpha^{(\mathrm{SHH})} [L_i^{(\mathrm{SHH})}][R_i^{(\mathrm{SHH})}] + p_i^{(\mathrm{SHH})} - d_L^{(\mathrm{SHH})} \left[L_i^{(\mathrm{SHH})}\right],$$

$$\frac{d\left[R_i^{(\mathrm{SHH})}\right]}{dt} = -\alpha^{(\mathrm{SHH})} \left[L_i^{(\mathrm{SHH})}\right]\left[R_i^{(\mathrm{SHH})}\right] - d_R^{(\mathrm{SHH})} \left[R_i^{(\mathrm{SHH})}\right],$$

$$\frac{d\left[C_i^{(\mathrm{SHH})}\right]}{dt} = \alpha^{(\mathrm{SHH})} \left[L_i^{(\mathrm{SHH})}\right]\left[R_i^{(\mathrm{SHH})}\right] - d_C^{(\mathrm{SHH})} \left[C_i^{(\mathrm{SHH})}\right],$$

$$\frac{d[\mathrm{F}_i]}{dt} = \alpha^{(\mathrm{F})} \mathrm{H}\left(1 + K_{\mathrm{SHH,F}} \left[C_i^{(\mathrm{SHH})}\right]\right) - d^{(\mathrm{F})} [\mathrm{F}_i],$$

$$\frac{d\left[L_i^{(\mathrm{BMP4})}\right]}{dt} = \mathcal{D}^{(\mathrm{BMP4})} \sum_{j=1}^{N} \Delta_{ij} \left[L_i^{(\mathrm{BMP4})}\right] + \alpha^{(\mathrm{BMP4})}\, H\big(1 + K_{\mathrm{F,BMP4}}[\mathrm{F}_i]\big) + p_i^{(\mathrm{BMP4})} - d_L^{(\mathrm{BMP4})} \left[L_i^{(\mathrm{BMP4})}\right].$$

To perturb the model, we inhibited the formation of bound SHH complex and production of FOXF1 due to SHH binding. Therefore, we set $\alpha^{(\mathrm{SHH})} \mapsto 10^{-3} * \alpha^{(\mathrm{SHH})}$, $\alpha^{(\mathrm{F})} \mapsto 10^{-3} * \alpha^{(\mathrm{F})}$, and $K_{\mathrm{SHH,F}} \mapsto 10^{-3} * K_{\mathrm{SHH,F}}$.

Inflow-driven patterning

We consider tissue-scale patterning driven by a spatially varying morphogen "gradient", in this case, due to SHH signaling[5]. This model is derived from studies of morphogen-driven gene regulatory mechanisms during neural tube development[5]. Here, the tissue pattern is described by the spatial expression of competing TFs—in this case, NKX2.2 (N), OLIG2 (O), PAX6 (P), and IRX3 (I)—that are either direct or indirect targets of bound SHH complex.

$$\frac{d\left[L_i^{(\mathrm{SHH})}\right]}{dt} = \mathcal{D}^{(\mathrm{SHH})} \sum_{j=1}^{N} \Delta_{ij} \left[L_i^{(\mathrm{SHH})}\right] - \alpha^{(\mathrm{SHH})} [L_i^{(\mathrm{SHH})}][R_i^{(\mathrm{SHH})}] + p_i^{(\mathrm{SHH})} - d_L^{(\mathrm{SHH})} \left[L_i^{(\mathrm{SHH})}\right],$$

$$\frac{d\left[R_i^{(\mathrm{SHH})}\right]}{dt} = -\alpha^{(\mathrm{SHH})} \left[L_i^{(\mathrm{SHH})}\right]\left[R_i^{(\mathrm{SHH})}\right] - d_R^{(\mathrm{SHH})} \left[R_i^{(\mathrm{SHH})}\right],$$

$$\frac{d\left[C_i^{(\mathrm{SHH})}\right]}{dt} = \alpha^{(\mathrm{SHH})} \left[L_i^{(\mathrm{SHH})}\right]\left[R_i^{(\mathrm{SHH})}\right] - d_C^{(\mathrm{SHH})} \left[C_i^{(\mathrm{SHH})}\right],$$

$$\frac{d[\mathrm{N}_i]}{dt} = \alpha^{(\mathrm{N})} \mathrm{H}\left(\frac{1 + K_{\mathrm{SHH,N}}\left[C_i^{(\mathrm{SHH})}\right]}{\big(1 + K_{\mathrm{O,N}}[\mathrm{O}]\big)^2 \big(1 + K_{\mathrm{P,N}}[\mathrm{P}]\big)^2 \big(1 + K_{\mathrm{I,N}}[\mathrm{I}]\big)^2}\right) - d^{(\mathrm{N})}[\mathrm{N}_i],$$

$$\frac{d[\mathrm{O}_i]}{dt} = \alpha^{(\mathrm{O})} \mathrm{H}\left(\frac{1 + K_{\mathrm{SHH,O}}\left[C_i^{(\mathrm{SHH})}\right]}{\big(1 + K_{\mathrm{N,O}}[\mathrm{N}]\big)^2 \big(1 + K_{\mathrm{I,O}}[\mathrm{I}]\big)^2}\right) - d^{(\mathrm{O})}[\mathrm{O}_i],$$

$$\frac{d[\mathrm{P}_i]}{dt} = \alpha^{(\mathrm{P})} \mathrm{H}\left(\frac{1}{\big(1 + K_{\mathrm{N,P}}[\mathrm{N}]\big)^2 \big(1 + K_{\mathrm{O,P}}[\mathrm{O}]\big)^2}\right) - d^{(\mathrm{P})}[\mathrm{P}_i],$$

$$\frac{d[\mathrm{I}_i]}{dt} = \alpha^{(\mathrm{I})} \mathrm{H}\left(\frac{1}{\big(1 + K_{\mathrm{N,I}}[\mathrm{N}]\big)^2 \big(1 + K_{\mathrm{O,I}}[\mathrm{O}]\big)^2}\right) - d^{(\mathrm{I})}[\mathrm{I}_i].$$

All model parameter values for the control condition are given in **Supplementary Table 2.** To perturb the model, we inhibited the formation of bound SHH complex, the activation of NKX2-2 and OLIG2 due to bound SHH complex formation, and the inhibitory parameters between all TFs. Therefore, we set $\alpha^{(\mathrm{SHH})} \mapsto 10^{-3} * \alpha^{(\mathrm{SHH})}$, $K_{\mathrm{SHH,N}} \mapsto 10^{-3} * K_{\mathrm{SHH,N}}$, $K_{\mathrm{SHH,O}} \mapsto 10^{-3} * K_{\mathrm{SHH,O}}$, $K_{\mathrm{O,N}} \mapsto 10^{-3} * K_{\mathrm{O,N}}$, $K_{\mathrm{P,N}} \mapsto 10^{-3} * K_{\mathrm{P,N}}$, $K_{\mathrm{I,N}} \mapsto 10^{-3} * K_{\mathrm{I,N}}$, $K_{\mathrm{N,O}} \mapsto 10^{-3} * K_{\mathrm{N,O}}$, $K_{\mathrm{I,N}} \mapsto 10^{-3} * K_{\mathrm{I,N}}$, $K_{\mathrm{N,P}} \mapsto 10^{-3} * K_{\mathrm{N,P}}$, $K_{\mathrm{O,P}} \mapsto 10^{-3} * K_{\mathrm{O,P}}$, $K_{\mathrm{N,I}} \mapsto 10^{-3} * K_{\mathrm{N,I}}$, and $K_{\mathrm{O,I}} \mapsto 10^{-3} * K_{\mathrm{O,I}}$.

Competition between intercellular inflows

We considered an example where two morphogens, BMP4 and SHH, compete to generate dorsoventral patterning, which is described by spatially varying expressions of TFs that are representative of the dorsal, intermediate, and ventral areas of the tissue, described by the variables, D, I, and V, respectively[6]. Here, both SHH and BMP4 diffuse and bind to their respective receptors, activating gene expression from the different spatial zones. We also model that BMP4 and SHH are produced at the dorsal and ventral end of the tissue, respectively, and diffuse towards the intermediate area. BMP4 signaling directly regulates D and I, while SHH regulates I and V, and D, I, and V coregulate each other. Therefore, the equations describing the patterning dynamics are given by:

$$\frac{d\left[L_i^{(\mathrm{BMP4})}\right]}{dt} = \mathcal{D}^{(\mathrm{BMP4})} \sum_{j=1}^{N} \Delta_{ij} \left[L_i^{(\mathrm{BMP4})}\right] - \alpha^{(\mathrm{BMP4})}[L_i^{(\mathrm{BMP4})}][R_i^{(\mathrm{BMP4})}] + p_i^{(\mathrm{BMP4})} - d_L^{(\mathrm{BMP4})} \left[L_i^{(\mathrm{BMP4})}\right],$$

$$\frac{d\left[R_i^{(\mathrm{BMP4})}\right]}{dt} = -\alpha^{(\mathrm{BMP4})} \left[L_i^{(\mathrm{BMP4})}\right]\left[R_i^{(\mathrm{BMP4})}\right] - d_R^{(\mathrm{BMP4})} \left[R_i^{(\mathrm{BMP4})}\right],$$

$$\frac{d\left[C_i^{(\mathrm{BMP4})}\right]}{dt} = \alpha^{(\mathrm{BMP4})} \left[L_i^{(\mathrm{BMP4})}\right]\left[R_i^{(\mathrm{BMP4})}\right] - d_C^{(\mathrm{BMP4})} \left[C_i^{(\mathrm{BMP4})}\right],$$

$$\frac{d\left[L_i^{(\mathrm{SHH})}\right]}{dt} = \mathcal{D}^{(\mathrm{SHH})} \sum_{j=1}^{N} \Delta_{ij} \left[L_i^{(\mathrm{SHH})}\right] - \alpha^{(\mathrm{SHH})}[L_i^{(\mathrm{SHH})}][R_i^{(\mathrm{SHH})}] + p_i^{(\mathrm{SHH})} - d_L^{(\mathrm{SHH})} \left[L_i^{(\mathrm{SHH})}\right],$$

$$\frac{d\left[R_i^{(\mathrm{SHH})}\right]}{dt} = -\alpha^{(\mathrm{SHH})}\left[L_i^{(\mathrm{SHH})}\right]\left[R_i^{(\mathrm{SHH})}\right] - d_R^{(\mathrm{SHH})}\left[R_i^{(\mathrm{SHH})}\right],$$

$$\frac{d\left[C_i^{(\mathrm{SHH})}\right]}{dt} = \alpha^{(\mathrm{SHH})}\left[L_i^{(\mathrm{SHH})}\right]\left[R_i^{(\mathrm{SHH})}\right] - d_C^{(\mathrm{SHH})}\left[C_i^{(\mathrm{SHH})}\right],$$

$$\frac{d[\mathrm{D}_i]}{dt} = \alpha^{(\mathrm{D})}\mathrm{H}\left(\frac{1 + K_{\mathrm{BMP4,D}}\left[C_i^{(\mathrm{BMP4})}\right]}{\left(1 + K_{\mathrm{I,D}}[\mathrm{I}]\right)^2\left(1 + K_{\mathrm{V,D}}[\mathrm{V}]\right)^2}\right) - d^{(\mathrm{D})}[\mathrm{D}_i],$$

$$\frac{d[\mathrm{I}_i]}{dt} = \alpha^{(\mathrm{I})}\mathrm{H}\left(\frac{\left(1 + K_{\mathrm{BMP4,I}}\left[C_i^{(\mathrm{BMP4})}\right]\right)\left(1 + K_{\mathrm{SHH,I}}\left[C_i^{(\mathrm{SHH})}\right]\right)}{\left(1 + K_{\mathrm{D,I}}[\mathrm{D}]\right)^2\left(1 + K_{\mathrm{V,I}}[\mathrm{V}]\right)^2}\right) - d^{(\mathrm{I})}[\mathrm{I}_i],$$

$$\frac{d[\mathrm{V}_i]}{dt} = \alpha^{(\mathrm{V})}\mathrm{H}\left(\frac{1 + K_{\mathrm{SHH,V}}\left[C_i^{(\mathrm{SHH})}\right]}{\left(1 + K_{\mathrm{D,V}}[\mathrm{D}]\right)^2\left(1 + K_{\mathrm{V,D}}[\mathrm{V}]\right)^2}\right) - d^{(\mathrm{V})}[\mathrm{V}_i].$$

All model parameter values for the control condition are given in **Supplementary Table 3.** To perturb the model, we inhibited the formation of both bound SHH complex and bound BMP4 complex, the activation of D and I due to binding of BMP4, the activation of I and V due to SHH, and all inhibitory parameters between D, I, and V. Therefore, we set $\alpha^{(\mathrm{SHH})} \mapsto 10^{-3} * \alpha^{(\mathrm{SHH})}$, $\alpha^{(\mathrm{BMP4})} \mapsto 10^{-3} * \alpha^{(\mathrm{BMP4})}$, $K_{\mathrm{BMP4,D}} \mapsto 10^{-3} * K_{\mathrm{BMP4,D}}$, $K_{\mathrm{BMP4,I}} \mapsto 10^{-3} * K_{\mathrm{BMP4,I}}$, $K_{\mathrm{SHH,I}} \mapsto 10^{-3} * K_{\mathrm{SHH,I}}$, $K_{\mathrm{SHH,V}} \mapsto 10^{-3} * K_{\mathrm{SHH,V}}$, $K_{\mathrm{I,D}} \mapsto 10^{-3} * K_{\mathrm{I,D}}$, $K_{\mathrm{V,D}} \mapsto 10^{-3} * K_{\mathrm{V,D}}$, $K_{\mathrm{D,I}} \mapsto 10^{-3} * K_{\mathrm{D,I}}$, $K_{\mathrm{V,I}} \mapsto 10^{-3} * K_{\mathrm{V,I}}$, $K_{\mathrm{D,V}} \mapsto 10^{-3} * K_{\mathrm{D,V}}$, and $K_{\mathrm{V,D}} \mapsto 10^{-3} * K_{\mathrm{V,D}}$.

Supplementary Results

Comparison to other methods

To better benchmark, we compared FlowSig's results to several methods that infer coordinated multicellular gene expression programs across multiple samples or conditions, including CellChat[7] alone, DIALOGUE[8], MOFAcellular[9] and MOFAtalk[9], scITD[10], MultiNicheNet[11], and Tensor-cell2cell[12]. We applied each of these methods to a common dataset by Kang et al.[13], who stimulated peripheral blood mononuclear (PBMC) cells sampled from lupus patients with Interferon-β. This data was sampled from eight patients, where each

patient sample was split into datasets (one control and one stimulated), yielding 16 individual samples in total.

We analyzed the version of Kang et al. dataset provided in the LIANA[14] tutorials, using the *“cell_abbr”* label for cell type annotations, the “condition” label to annotate stimulation condition, and the “sample” label to separate the 16 individual samples (8 patients, before and after Interferon-β stimulation). Cells from the dataset expressing fewer than 200 genes and genes expressed in fewer than 3 cells were removed, yielding a dataset containing 24, 562 cells and 15, 701 genes. We then normalized gene expression counts so that each cell had 10, 000 total counts and log-transformed the normalized expression values with a pseudocount of 1. In this dataset, there are eight total cell types: CD14+ monocytes (CD14), CD4 T cells (CD4T), dendritic cells (DCs), Natural Killer cells (NK), CD8 T cells (CD8T), B cells (B), FCGR3A+ Monocytes (FGR3), and Megakaryocytes (Mega). We note that Mega cells were not present across all samples.

To generate input for FlowSig, we applied CellChat to each condition-specific dataset separately (control and stimulated), using the “cell_abbr” annotations as the group labels. FlowSig constructed 20 GEMs using pyLIGER from the raw, unnormalized gene expression counts. We then only retained signal inflow and signal outflow variables which were sufficiently differentially flowing between the control and stimulated condition ($p.adj < 0.05$, $\log(FC) > 0.5$), yielding 53 total variables (13 signal inflow, 20 GEMs, 20 signal outflow variables). FlowSig inferred the intercellular flow network across 500 bootstrap realizations and removed edges with a bootstrapped edge frequency less than 0.7. The resulting intercellular flow network contained 6 signal inflow variables, 20 GEM variables, and 12 signal outflow nodes. FlowSig inferred that intercellular flows were driven by inflow into C3AR1 (via C3), C5AR1 (via HC), CCR1 (via CCL3, CCL5, CCL7, CCL8), CXCR3 (via CXCL9, CXCL10, CXCL11), IL7R+IL2RG (via IL7), and PLAUR (via PLAUR), and subsequent outflow from CCL4, CXCL10, CXCL11, CXCL2, CXCL3, FASLG, GZMA, IL1B, LGALS9, TNFSF10, TNFSF13B, and TNFSF14.

To examine the direct CellChat output, we searched for inflow-to-outflow relationships encoded by the pair of ligand-receptor interactions, $(L_1 \sim R_1,\ L_2 \sim R_2)$, for which there existed a cell type triplet, (A, B, C), such that A communicates with B via the ligand-receptor interaction $L_1 \sim R_1$ and B communicates with C via $L_2 \sim R_2$. Analysis of CellChat output implied that there are 7, 209 inflow-to-out-flow relationships across either control or stimulated condition, with 3, 382 flows shared across both conditions, 1, 687 flows unique to the control condition, and 2, 140 flows unique to the stimulated condition. We then restricted output to only paracrine intercellular flows, i.e., where A, B, and C are all different cell types but the ligand-receptor interactions may be the same. After removing flows that did not satisfy this criteria, CellChat output implies a total of 6, 886 intercellular flows, with 3, 167 shared across both conditions, 1, 511 flows unique to the control condition, and 2, 208 flows unique to the stimulated condition.

We applied DIALOGUE to the Kang et al. dataset. DIALOGUE uses penalized matrix decomposition and multileveling modeling to construct latent multicellular programs from scRNA-seq or ST data. DIALOGUE identified four multicellular programs, MCP1 through 4. To ensure DIALOGUE ran correctly, we removed Megakaryocytes from the analysis as they were not present across all samples. DIALOGUE inferred that MCP1 was significantly associated with CD14 cells, CD8T cells, and B cells; MCP2 was most associated with CD4T and CD8T cells; MCP3 was most associated with DCs and FGR3 cells; and MCP4 was most significantly associated with B and FGR3 cells. DIALOGUE also inferred that stimulation induced upregulation of the genes CFP and COA6 in CD14 cells, upregulation of CCL4, CCL5, CD2, CD3D, CD3E, CD3G, CD52, CD88, FGFBP2, GIMAP7, GZMH, HLA-DPA1, HLA-DPB1, MALAT1, NKG7, SH3BGRL3, TBC1D10C, TRAF3IP3, VAMP5, and ZNF683 in CD8T cells, and downregulation of SRGN in CD8T cells.

We also applied scITD, which uses a tensor decomposition approach to identify GEMs that account for between-sample variation. To ensure scITD ran correctly, we removed Megakaryocytes from the analysis as they were not present across all samples. We constructed the scITD tensor using all default parameters. We determined the number of

samples-specific factors in which to decompose the Kang et al. by using the scITD function *determine_ranks_tucker. scITD* identified two sample-specific factors, Factor 1 and Factor 2. Factor 1 was significantly associated with the stimulated condition and was significantly enriched across all eight stimulated samples. Significantly associated genes with Factor 1 included CXCL11, APOBEC3A, CXCL10, TNFSF10, MX1, IFIT1, IFIT3, IFI6, ISG15. scITD also inferred ligand-receptor interactions significantly associated with Factor 1, including CCL2 – CCR5, CCL2 – CCR1, CCL8 – CCR1, CCL8 – CCR5, CXCL10 – CXCR3, CXCL11 – CXCR3, HLA-E – KLRC1, HLA-E – KLRC2, HLA-E – KLRD1, TNFSF10 – TNFRSF10A, TNSF10 – TNFRSF10B, TNFSF10 – TNFRSF10D, TNFSF13B – TNFRSF13BC, TNFSF13B – TNFRSF13C, TNSF14 – TNFRSF14, ILRN – IL1R2, IL14 – IL2RG, IL15 – IL14RA, ANXA1 – FPR3, IL15 – IL2RB, CXCL9 – CXCR3, CD40 – CD40LG, HLA-E – CD8A, ITGB2 – ICM1, SPN – SIGLEC1, TNSF13B – TNFRSF13B, PTPRC – CD22, FASLG – FAS, ALOX5AP – ALOX5, ICAM2 – ITGB2, SERPING1 – LRP1, TNSF18 – TNFRSF18, HLA-A – CD8A. Of these, involved in intercellular flows

We applied MOFAcellular to the Kang et al. dataset, following the default workflow. For preprocessing, MOFAcellular pseudobulked the unnormalized gene expression counts by cell type, keeping track of condition and patient (not sample). We only retained cell types present in at least three cell types and with a sample size of at least 25, and with at least 50 total counts. As a result of these filters, MOFAcellular removed Mega cells from analysis. MOFAcellular then normalized and log-transformed the pseudobulked data per “view” (cell type). For each cell type, we only considered genes that were differentially expressed. We then decomposed the transformed pseudobulked data into five factors. Using a t-test, MOFAcellular identified that Factor 1 was significantly associated with stimulation condition and significantly downregulated in stimulated samples The highest scoring genes for Factor 1 were TNFSF10, RSAD2, OASL, MX1, LY6E, ISG20, ISG15, IL1RN, IFIT3, IFIT2, IFIT1, IFI6, CXCL11.

We also applied MOFAtalk to the Kang et etl. dataset. MOFAtalk used LIANA[15] to infer ligand-receptor interactions for each sample. We then aggregated the ligand-receptor scores

(*'magnitude_rank'* calculated by LIANA*)* across interacting cell type pairs, keeping condition and patient as covariates. We only aggregated samples for which there were at least 10 inferred ligand-receptor interactions and only retained ligand-receptor interactions which were present in at least 30% of samples. We then decomposed the aggregated ligand-receptor data into five latent factors. We used a t-test to determine that Factor 1 was significantly associated with stimulation condition and upregulated for the stimulated Interferon-β condition. MOFAtalk identified several ligand-receptor interactions that were significantly associated with Factor 1, including TNFSF13B – CD40, CCL2 – CCR5, CCL8 – CCR5, CCL2 – CCR1, HLA-DQB1 – LAG3, HLA-DQA1 – LAG3, CCL5 – CCRL2, LGALS1 – CD69, LGALS3 – LAG3, TNFSF13B – HLA-DPB1, HLA-DPB1 – LAG3, CCL8 – CCR1, HLA-DPA1 – LAG3, HLA-DRB1 – LAG3, HLA-DRA – LAG3.

To apply MultiNicheNet to the Kang et al. dataset, we first removed Megakaryocytes from analysis. MultiNicheNet uses muscat[16] to perform differential expression analysis of the pseudobulked (by sample) counts with respect to stimulation condition. For each potential signal receiver cell type, MultiNicheNet performs ligand-target analysis of the differentially expressed genes. MultiNicheNet identified significantly upregulated ligand-receptor interactions in the stimulated condition, including MDK – ITGA4, IL15 – IL15RA, FASLG – FAS, SLAMF7 – SLAMF7, SIRPA – CD47, SIGLEC1 – CD47, LGALS9 – CD47, IL15 – IL15RA, CXCL11 – CCR5, CD80 – CD274, CD47 – SIGLEC, CD274 – CD80, CCL8 – CCR5, CCL8 – CCR1, CCL7 – CCR1, CCL7 – CCR5, CCL4 – CCR5, CCL4 – CCR1, CCL3 – CCR5, CCL3 – CCR1, CCL2 – CCR5, CCL2 – CCR1, SPN – SIGLEC1. To construct the intercellular regulatory network, we only retained the top 50 ligand-receptor interactions, retaining edges in the network such that the target genes of the receiver cell type correlated significantly with the ligand genes expressed by the signal cell type. MultiNicheNet identified that CCL2 in CD14 cells was upregulated by many other ligands and cell types, including autocrine regulation by other CD14 cells via CCL3, CCL7, CCL8, CXCL11, IL15, CCL4, SLAMF7, LGALS9, SIRPA, and CD45, and CD80; paracrine regulation by DCs via SLAMF7, SPN, CD47, CXCL11, and LGALS9; and by FGR3 cells via CXCL11, IL15, and LGALS9. Another outflow variable, CCL3

in CD14 cells, was inferred to be upregulated by LGALS9 secreted by other CD14 cells, DCs, and FGR3 cells, IL15 cells in FGR3 cells, and CCL8, IL15, and CD247 by other CD14 cells. The other significant hub was FAS in DCs, which was upregulated by SLAMF7 from FGR3, DCs and CD14 cells, FASLG from NK, LGALS9 from DCs and B cells, SIGLEC1 from CD14 cells, and LGALS9 from CD14, FGR3, DCs.

Before applying Tensor-cell2cell, we first inferred ligand-receptor interactions using LIANA. Tensor-cell2cell then constructed a ligand-receptor interaction tensor with respect to samples. We then decomposed the tensor into six factors. Using a Student's t-test, Tensor-cell2cell inferred that Factor 3, Factor 5, and Factor 6 were most significantly associated with stimulation condition. Of these three factors, Factor 3 was downregulated in stimulated samples, while Factors 5 and Factor 6 were upregulated in stimulated samples. Clustering Tensor-cell2cell ligand-receptor interactions revealed that Factor 3 was most enriched for S100A4 – CCR5, GNAI2 – CCR5, GRN – TNFRSF1A, GRN – TNFRSF1B, S100A9 – CD36, CD14 – ITGB2, S100A9 – CD68, S100A9 – ITGB2, S100A8 – ITGB2, S100A8 – CD68, VCAN – ITGB1, CD14 – ITGB1, S100A8 – CD36, VCAN – TLR2, HEBP1 – FRP3, LILRB4 – LAIR1, GNAI2 – C5AR1, LGALS3 – ENG, GNAI2 – S1PR4, VCAN – CD44, THBS1 – CD47, ICAM1 – IL2RG, SIRPA – CD47, PSEN1 – CD44. Factor 5 was most enriched for HLA-A – LILRB1, HLA-B – LILRB1, HLA-C – LILRB1, B2M – LILRB1, HLA-F – LILRB1, ARF6 – SMAP1, HLA-DPB1 – LAG3, HLA-DPA1 – LAG3, CALM1 – PTPRA, LGALS9 – HAVCR2, CCL8 – CCR5, CCL8 – CCR1, CALM1 – FAS, CCL3 – CCR1, CCL3 – CCR5, CCL2 – CCR1, CCL2 – CCR5, CCL4 – CCR1, CCL4 – CCR5, and TNFSF13B – CD40, TNFSF13B – HLA-DPB1, LGALS9 – CD44, LGALS9 – PTPRC, LGALS9 – CD47. Factor 6 was most enriched for ICAM1 – IL2RG, SIRPA – CD47, PSEN1 – CD44, TGFB1 – CXCR4, S100A8 – CD69, TNFSF13B – HLA-DPB1, LGALS9 – CD44, LGALS9 – PTPRC, LGALS9 – CD47, LGALS1 – CD69, TIMP1 – CD63, LGALS1 – PTPRC, HMGB1 – CXCR4, PKM – CD44, VIM – CD44.

There are many differences between FlowSig and these other methods However, the methods that analyze ligand-receptor activity (MOFAtalk, MultiNicheNet, Tensor-cell2cell, scITD) primarily extract coordinated programs of pairwise ligand-receptor activities. Other

differences stem from not focus on gene expression without distinguishing between signaling roles (DIALOGUE, scITD, MOFAcellular) or ligand-receptor interaction scores (MOFAtalk, MultiNicheNet, Tensor-cell2cell) or the use of different preexisting databases used by other methods (for example, FlowSig primarily uses CellChat while MOFA and Tensor-cell2cell use OmniPath). Aside from MultiNicheNet, FlowSig is the only method that constructs a directed network and integrates intercellular information (via ligand-receptor interactions) with intracellular information via GEMs for FlowSig and known receptor-TF relations for FlowSig and MultiNicheNet).

Implementing FlowSig with different cell-cell communication models and GEM construction methods

To demonstrate the flexibility of the FlowSig framework, we also considered implementations where we used input from either a different cell-cell communication model or GEM construction method. Therefore, we also implemented one FlowSig workflow where cell-cell communication input was generated from CellPhoneDB[17] instead of CellChat, and one workflow where GEMs were constructed using cNMF[18] instead of pyLIGER. Both workflows were applied to the Kang et al. PBMC dataset.

To implement the FlowSig workflow with CellPhoneDB, we used the "DEG-based" CellPhoneDB method to infer significant ligand-receptor intearctions for each Kang et al. condition. In order to analyze the data using CellPhoneDB, for each condition-specific dataset, we constructed a list of the differentially expressed genes for each cell type. From this list, we also extracted a list of differentially expressed TFs for each cell type. CellPhoneDB then inferred ligand-receptor actions between cell type ("cell_abbr") pairs. We then only retained ligand-receptor interactions involving secreted diffusible ligands and directed ligand-receptor binding. When constructing the flow expression matrix, only the way we construct signal inflow variables changes when using CellPhoneDB. Instead of reweighting receptor gene expression by "known" downstream TFs from the OmniPath database[14], we take advantage of

CellPhoneDB's CellSign to infer which ligand-receptor interactions are connected to active downstream TFs. Therefore, we reweight receptor gene expression by the expression of active TFs inferred from CellPhoneDB. All other FlowSig analysis was the same as when we used CellChat's results as input.

To implement FlowSig with cNMF, we used CellChat's results as input. To applying cNMF to the unnormalized Kang et al. gene expression counts data, we first calculated the top 2, 000 highly variable genes (using *Scanpy*'s default dispersion-based method). To determine the optimal number of cNMF GEMs, K, we ran cNMF over a range of K values, generating 200 cNMF replicates for each value of K. Using cNMF guidelines, we then selected the value of K with the highest average stability, yielding $K = 8$ as the optimal number of cNMF GEMs. All other analysis was the same as the default FlowSig workflow.

Robustness of FlowSig to different input methodologies

We investigated how FlowSig's results changed when we either 1) used output from a different cell-cell communication method to construct the signal inflow and outflow variables or 2) used a different method to construct the intracellular GEMs. In the first comparison, we compared output from CellChat[19] *vs.* output from CellPhoneDB[17], another popular cell-cell communication inference method. In the second comparison, we compared GEMs constructed from pyLIGER[20] to GEMs constructed from cNMF[18]. We performed both comparisons using the dataset of stimulated PBMCs generated by Kang et al. [13] (see "Implementing FlowSig with different cell-cell communication models and GEM construction methods" in **Supplementary Notes**).

We observed that the sets of signal inflow and outflow nodes inferred by FlowSig to drive intercellular flows do change somewhat if one uses cNMF rather than pyLIGER for GEM construction (**Supplementary Figure 7b**). These differences may arise because the GEMs constructed by pyLIGER are more enriched for stimulation condition (**Supplementary Figure 1a**), while the GEMs constructed by cNMF are more enriched for cell type (**Supplementary**

Figure 7c). However, when we examined the intercellular flows between common signal inflow and outflow variables, we found that, for every signal outflow variable, FlowSig infers directed paths from signal inflow variables through GEMs that are enriched for the same TFs. For example, FlowSig and pyLIGER inferred that inflow through C5AR1 drives CXCL10 outflow via GEM-1, GEM-10, GEM-11, and GEM-20, while FlowSig and cNMF inferred that inflow through C5AR1 drives CXCL10 outflow via one module, cNMF-6. Calculating the intersection between the top genes contributing to these GEM sets revealed that there were seven TFs shared—notably, CREM in GEM-1 and cNMF-6 (**Supplementary Figure 1c, 7c**). This suggests that even when using a different GEM method, FlowSig will still infer intercellular flows through GEMs that are enriched for the same regulatory TFs.

References

1. Burkhardt, D. B. *et al.* Quantifying the effect of experimental perturbations at single-cell resolution. *Nat Biotechnol* **39**, 619–629 (2021).
2. Hie, B., Bryson, B. & Berger, B. Efficient integration of heterogeneous single-cell transcriptomes using Scanorama. *Nat Biotechnol* **37**, 685–691 (2019).
3. Muraro, M. J. *et al.* A Single-Cell Transcriptome Atlas of the Human Pancreas. *Cell Syst* **3**, 385-394.e3 (2016).
4. Bohnenpoll, T. *et al.* A SHH-FOXF1-BMP4 signaling axis regulating growth and differentiation of epithelial and mesenchymal tissues in ureter development. *PLoS Genet* **13**, e1006951 (2017).
5. Briscoe, J. & Small, S. Morphogen rules: design principles of gradient-mediated embryo patterning. *Development* **142**, 3996–4009 (2015).
6. Zagorski, M. *et al.* Decoding of position in the developing neural tube from antiparallel morphogen gradients. *Science (1979)* **356**, 1379–1383 (2017).
7. Jin, S. *et al.* Inference and analysis of cell-cell communication using CellChat. *Nat Commun* **12**, (2021).
8. Jerby-Arnon, L. & Regev, A. DIALOGUE maps multicellular programs in tissue from single-cell or spatial transcriptomics data. *Nat Biotechnol* **40**, 1467–1477 (2022).
9. Flores, R. O. R., Lanzer, J. D., Dimitrov, D., Velten, B. & Saez-Rodriguez, J. Multicellular factor analysis of single-cell data for a tissue-centric understanding of disease. *Elife* **12**, (2023).
10. Mitchel, J. *et al.* Tensor decomposition reveals coordinated multicellular patterns of transcriptional variation that distinguish and stratify disease individuals. *bioRxiv* 1–47 (2022) doi:10.1101/2022.02.16.480703.
11. Browaeys, R. *et al.* MultiNicheNet: a flexible framework for differential cell-cell communication analysis from multi-sample multi-condition single-cell transcriptomics data. *bioRxiv* 1–64 (2023) doi:10.1101/2023.06.13.544751.
12. Armingol, E. *et al.* Context-aware deconvolution of cell–cell communication with Tensor-cell2cell. *Nat Commun* **13**, (2022).
13. Kang, H. M. *et al.* Multiplexed droplet single-cell RNA-sequencing using natural genetic variation. *Nat Biotechnol* **36**, 89–94 (2018).
14. Türei, D. *et al.* Integrated intra- and intercellular signaling knowledge for multicellular omics analysis. *Mol Syst Biol* **17**, 1–16 (2021).
15. Dimitrov, D. *et al.* Comparison of methods and resources for cell-cell communication inference from single-cell RNA-Seq data. *Nat Commun* **13**, 3224 (2022).
16. Crowell, H. L. *et al.* muscat detects subpopulation-specific state transitions from multi-sample multi-condition single-cell transcriptomics data. *Nat Commun* **11**, 6077 (2020).
17. Garcia-Alonso, L. *et al.* Single-cell roadmap of human gonadal development. *Nature* **607**, 540–547 (2022).
18. Kotliar, D. *et al.* Identifying gene expression programs of cell-type identity and cellular activity with single-cell RNA-Seq. *Elife* **8**, (2019).
19. Jin, S. *et al.* Inference and analysis of cell-cell communication using CellChat. *Nat Commun* 1–20 (2021) doi:10.1101/2020.07.21.214387.
20. Lu, L. & Welch, J. D. PyLiger: scalable single-cell multi-omic data integration in Python. *Bioinformatics* **38**, 2946–2948 (2022).

21. Sachs, K., Perez, O., Pe'er, D., Lauffenburger, D. A. & Nolan, G. P. Causal protein-signaling networks derived from multiparameter single-cell data. *Science (1979)* **308**, 523–529 (2005).
22. Chen, X. *et al.* An individualized causal framework for learning intercellular communication networks that define microenvironments of individual tumors. *PLoS Comput Biol* **18**, e1010761 (2022).
23. Fischer, D. S., Schaar, A. C. & Theis, F. J. Modeling intercellular communication in tissues using spatial graphs of cells. doi:10.1038/s41587-022-01467-z.
24. Arnol, D., Schapiro, D., Bodenmiller, B., Saez-Rodriguez, J. & Stegle, O. Modeling Cell-Cell Interactions from Spatial Molecular Data with Spatial Variance Component Analysis. *Cell Rep* **29**, 202-211.e6 (2019).

Supplementary Tables

Method	Input	Methodology	Output
Sachs et al.[21]	Multi-condition single-cell phosphoprotein expression from flow cytometry	Score-based Bayesian network structure inference algorithm	Directed acyclic graph encoding direct dependencies between protein variables
Chen et al.[22]	Multi-sample scRNA-seq	NHDP to construct GEMs; FCI algorithm applied to GEM variables	Partial ancestral graph encoding direct dependencies and ambiguous relations obscured by confounding
NCEM[23]	Spatial transcriptomics data with cell type labels and batch assignment labels	Graph neural networks to model gene expression as linear function of expression in spatial neighborhood	Matrix of effects of sender cell types on receiver cell types
SVCA[24]	Spatial proteomics data or spatial transcriptomics	Gaussian process modeling covariance as linear combination of intrinsic effects, cell-cell interactions, and environmental effects	Decomposition of explained variance for individual genes
DIALOGUE[8]	scRNA-seq or spatial transcriptomics with covariate metadata (condition, celltypes)	Penalized matrix decomposition and multilevel modeling	Multicellular programs describing coordinated gene expression between two or more cell types
scITD[10]	Multi-sample scRNA-seq with metadata	Per-sample pseudobulking; 3D tensor (sample-gene-cell type); Tucker tensor decomposition	Factors significantly associated with metadata

MOFAcellular[9]	Multi-sample scRNA-seq with condition and cell type labels	Per-sample pseudobulking by cell type; factor decomposition using Bayesian framework	Sample-specific factors associated with metadata; gene-driven contributions to factors
MOFAtalk[9]	Multi-sample scRNA-seq ligand-receptor interaction scores between cell types	Factor decomposition using Bayesian framework	Sample-specific factors associated with metadata; interaction-driven contributions to factors for cell types
MultiNicheNet[11]	Multi-sample scRNA-seq with condition and cell type labels	Per-sample pseudobulking by cell type; differential expression; ligand activity prediction	Directed cell-type-specific intercellular communication networks
Tensor-cell2cell[12]	Multi-sample scRNA-seq ligand-receptor interaction scores between cell types	Construction of ligand-receptor interaction tensor; tensor rank decomposition	Factors associated with conditions; factor contributions driven by ligand-receptor interactions between cell types
**FlowSig**	Multi-condition scRNA-seq or spatial transcriptomics; output from cell-cell communication method	GEM construction using matrix factorization; inflow and outflow variable construction; UT-IGSP algorithm and bootstrapping to infer dependencies between inflows, GEMs, and outflows	Completed partially directed acyclic graph encoding directed dependencies from inflows to GEMs, to outflows, and undirected dependencies between GEMs

Supplementary Table 1: Summary of FlowSig’s features and its similarities and differences with other comparable methods.

Parameter	Interpretation	Value
$\mathcal{D}^{(\mathrm{SHH})}$	Diffusivity of SHH ligand	100
$\alpha^{(\mathrm{SHH})}$	Binding rate of SHH ligand	2
$p^{(\mathrm{SHH})}$	Maximum production rate of SHH ligand	0.1
$d_L^{(\mathrm{SHH})}$	Degradation rate of free SHH ligand	0.1
$d_R^{(\mathrm{SHH})}$	Degradation rate of free SHH receptor	0
$d_C^{(\mathrm{SHH})}$	Degradation rate of bound SHH complex	0
$\alpha^{(\mathrm{F})}$	Production rate of FOXF1	2
$K_{\mathrm{SHH,F}}$	Promotion rate of FOXf1 due to bound SHH complex	25
$d^{(\mathrm{F})}$	Degradation rate of FOXF1	2
$\mathcal{D}^{(\mathrm{BMP4})}$	Diffusivity of BMP4 ligand	100
$\alpha^{(\mathrm{BMP4})}$	Production rate of BMP4 ligand due to FOXF1 conversion	2
$K_{\mathrm{F,BMP4}}$	Promotion rate of BMP4 due to FOXF1	10
$p^{(\mathrm{BMP4})}$	Maximum autonomous production rate of BMP4 ligand	0.1
$d_L^{(\mathrm{BMP4})}$	Degradation rate of BMP4 ligand	0.2

Supplementary Table 2: Model parameter values for simulation model of SHH-induced outflow of BMP4.

Parameter	Interpretation	Value
$\mathcal{D}^{(\mathrm{SHH})}$	Diffusivity of SHH ligand	100
$\alpha^{(\mathrm{SHH})}$	Binding rate of SHH ligand	2
$p^{(\mathrm{SHH})}$	Maximum production rate of SHH ligand	0.1
$d_L^{(\mathrm{SHH})}$	Degradation rate of free SHH ligand	0.1
$d_R^{(\mathrm{SHH})}$	Degradation rate of free SHH receptor	0
$d_C^{(\mathrm{SHH})}$	Degradation rate of bound SHH complex	0
$\alpha^{(\mathrm{N})}$	Production rate of NKX2.2	2
$K_{\mathrm{SHH,N}}$	Promotion rate of NKX2.2 due to bound SHH complex	375
$K_{\mathrm{O,N}}$	Inhibition rate from OLIG2 to NKX2.2	27
$K_{\mathrm{P,N}}$	Inhibition rate from PAX6 to NKX2.2	5
$K_{\mathrm{I,N}}$	Inhibition rate from IRX3 to NKX2.2	76
$d^{(\mathrm{N})}$	Degradation rate of NKX2.2	2
$\alpha^{(\mathrm{O})}$	Production rate of OLIG2	2
$K_{\mathrm{SHH,O}}$	Promotion rate from bound SHH complex to OLIG2	20
$K_{\mathrm{N,O}}$	Inhibition rate from NKX2.2 to OLIG2	60
$K_{\mathrm{I,O}}$	Inhibition rate from IRX3 to NKX2.2	60
$d^{(\mathrm{O})}$	Degradation rate of OLIG2	2
$\alpha^{(\mathrm{P})}$	Production rate of PAX6	2
$K_{\mathrm{N,P}}$	Inhibition rate from NKX2.2 to PAX6	25
$K_{\mathrm{O,P}}$	Inhibition rate from OLIG2 to PAX6	2
$d^{(\mathrm{P})}$	Degradation rate of PAX6	2
$\alpha^{(\mathrm{I})}$	Production rate of IRX3	2
$K_{\mathrm{N,I}}$	Inhibition rate from NKX2.2 to IRX3	75
$K_{\mathrm{O,I}}$	Inhibition rate from OLIG2 to IRX3	15
$d^{(\mathrm{I})}$	Degradation rate of IRX3	2

Supplementary Table 3*:* Model parameter values for simulation model of SHH-induced neural tube patterning.

Parameter	Interpretation	Value
$\mathcal{D}^{(\mathrm{BMP4})}$	Diffusivity of BMP4 ligand	200
$\alpha^{(\mathrm{BMP4})}$	Binding rate of BMP4 ligand	2
$p^{(\mathrm{BMP4})}$	Maximum production rate of BMP4 ligand	0.1
$d_L^{(\mathrm{BMP4})}$	Degradation rate of free BMP4 ligand	0.1
$d_R^{(\mathrm{BMP4})}$	Degradation rate of free BMP4 receptor	0
$d_C^{(\mathrm{BMP4})}$	Degradation rate of bound BMP4 complex	0
$\mathcal{D}^{(\mathrm{SHH})}$	Diffusivity of SHH ligand	200
$\alpha^{(\mathrm{SHH})}$	Binding rate of SHH ligand	2
$p^{(\mathrm{SHH})}$	Maximum production rate of SHH ligand	0.1
$d_L^{(\mathrm{SHH})}$	Degradation rate of free SHH ligand	0.1
$d_R^{(\mathrm{SHH})}$	Degradation rate of free SHH receptor	0
$d_C^{(\mathrm{SHH})}$	Degradation rate of bound SHH complex	0
$\alpha^{(\mathrm{D})}$	Production rate of D	2
$K_{\mathrm{BMP4,D}}$	Promotion rate from bound BMP4 complex to D	10
$K_{\mathrm{I,D}}$	Inhibition rate from I to D	10
$K_{\mathrm{V,D}}$	Inhibition rate from V to D	15
$d^{(\mathrm{D})}$	Degradation rate of D	2
$\alpha^{(\mathrm{I})}$	Production rate of I	2
$K_{\mathrm{BMP4,I}}$	Promotion rate from bound BMP4 complex to I	10
$K_{\mathrm{SHH,I}}$	Promotion rate from bound SHH complex to I	25
$K_{\mathrm{D,I}}$	Inhibition rate from D to I	15
$K_{\mathrm{V,I}}$	Inhibition rate from V to I	15
$d^{(\mathrm{I})}$	Degradation rate of I	2
$\alpha^{(\mathrm{V})}$	Production rate of V	2

$K_{\mathrm{SHH,V}}$	Promotion rate from bound SHH complex to V	25
$K_{\mathrm{I,V}}$	Inhibition rate from I to V	10
$K_{\mathrm{D,V}}$	Inhibition rate from D to V	10
$d^{(\mathrm{V})}$	Degradation rate of V	2

Supplementary Table 4: Model parameter values for simulation model of competition between SHH and BMP4 inflow to generate dorsoventral patterning.

Supplementary Figures

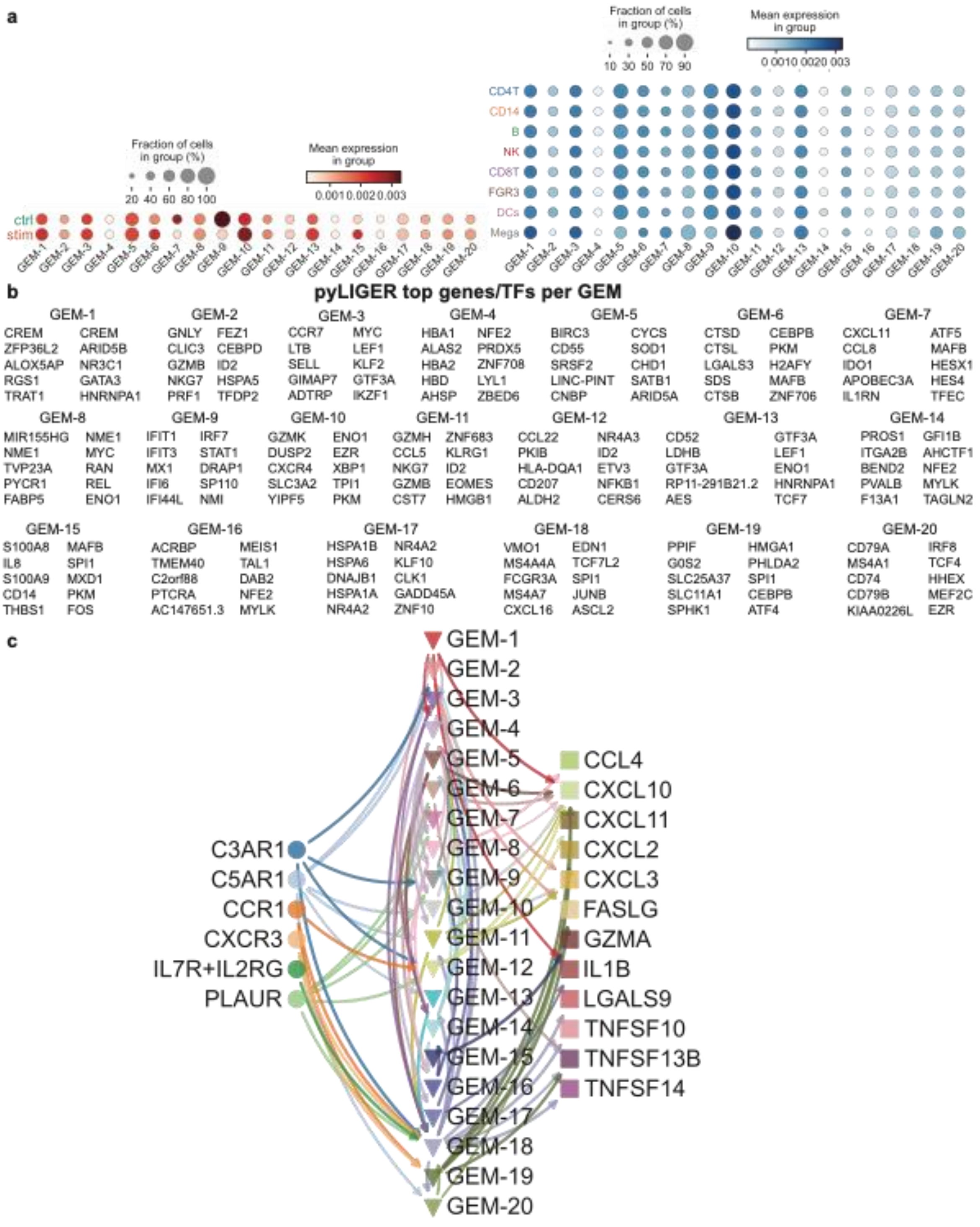

Supplementary Figure 1: FlowSig applied to the Kang *et al.* PBMC dataset. a Construction of 20 GEMs using pyLIGER, which are more enriched for stimulation condition than cell type. **b** The top five contributing genes and TFs for each GEM. For each GEM, the left column contains the top five overall genes, while the right column contains the top five TFs. **c** The directed intercellular flow network inferred by FlowSig.

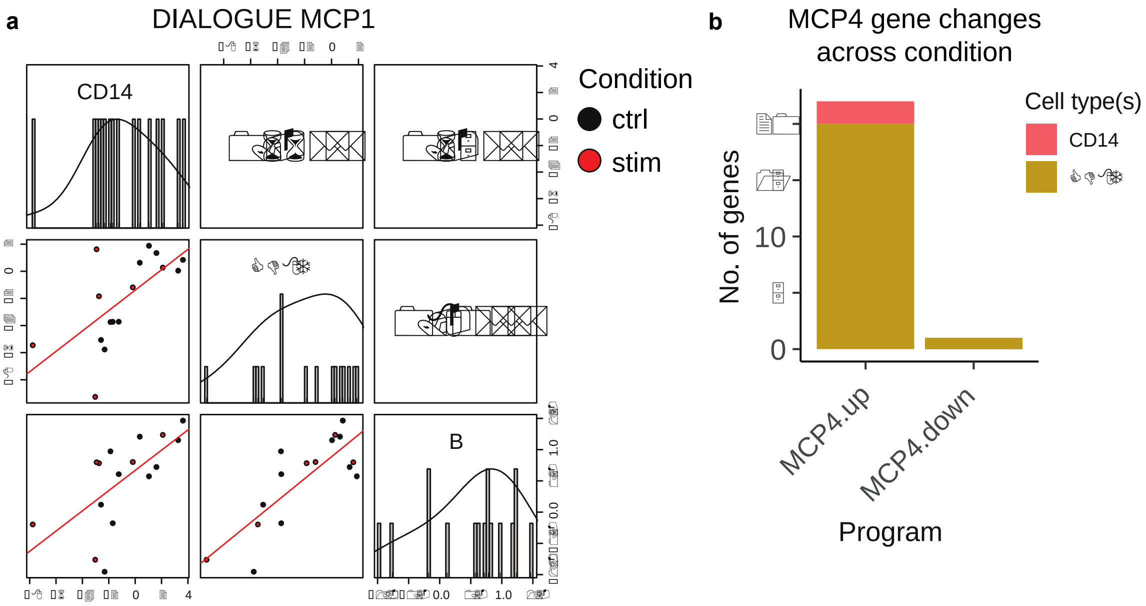

Supplementary Figure 2: DIALOGUE applied to the Kang *et al.* PBMC dataset. a MCP1 suggests coordinated expression between CD14, CD8T, and B cells. **b** MCP4 is enriched for differentially up- and down-regulated genes in CD14 and CD8T cells due to stimulation.

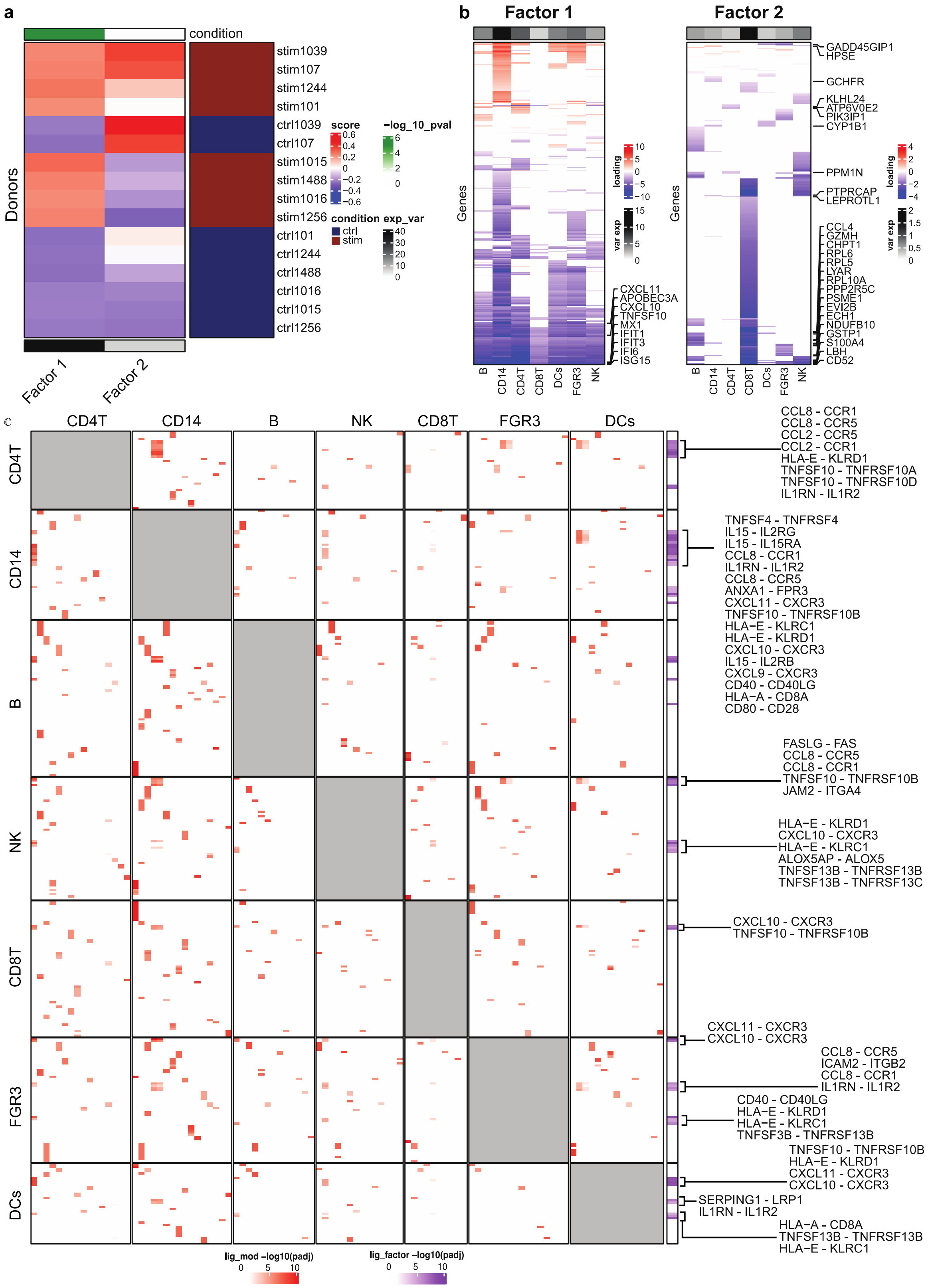

Supplementary Figure 3: scITD applied to the Kang *et al.* PBMC dataset. a The two sample-specific factors inferred by scITD. Factor 1 is significantly associated with stimulation. **b** The top genes associated with each factor. **c** Inferred ligand-receptor interactions associated with Factor 1.

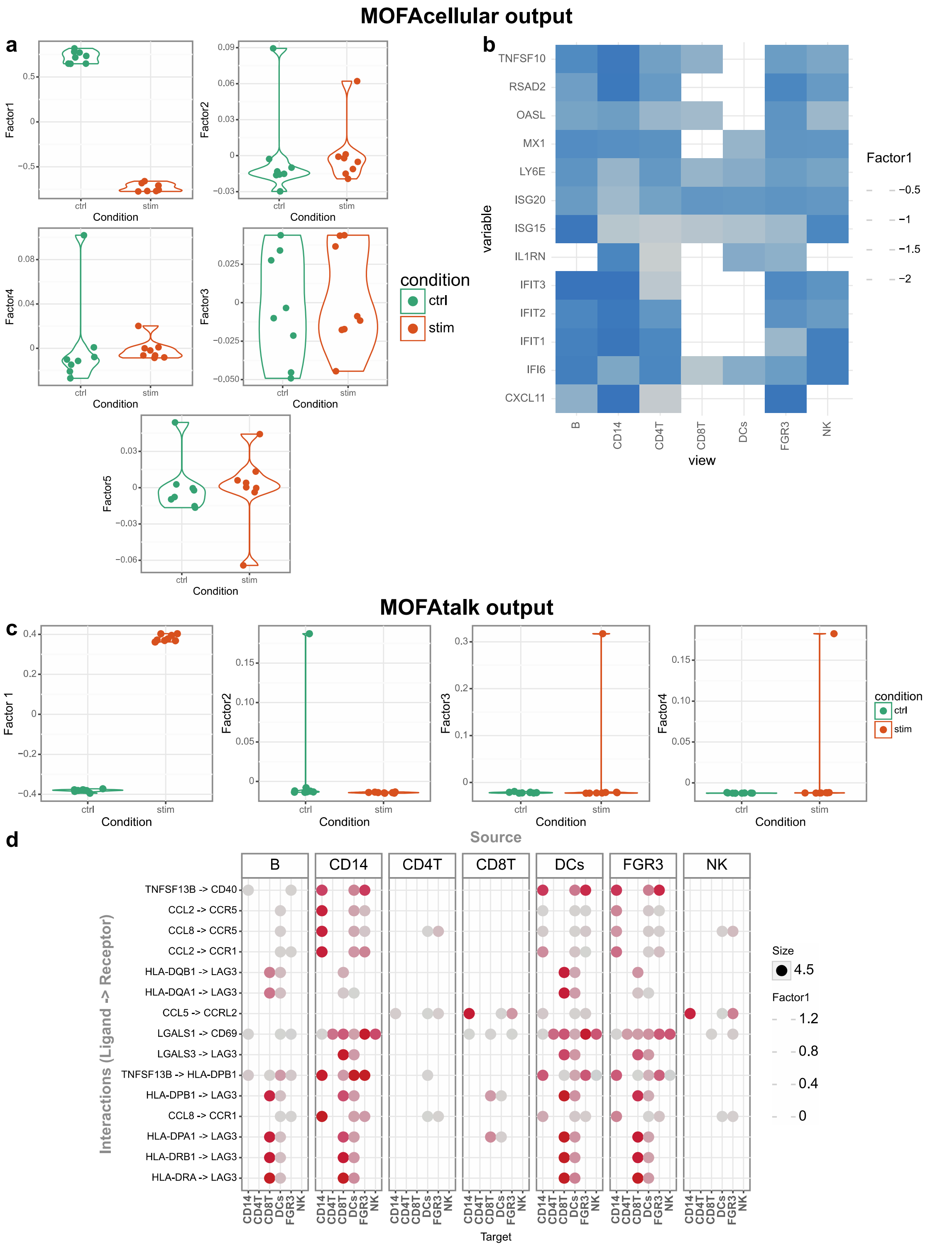

Supplementary Figure 4: MOFAcellular and MOFAtalk applied to the Kang *et al.*[13] PBMC dataset. a–b MOFAcellular applied to gene expression counts. **a** The five latent factors inferred by MOFAcellular. Only Factor1 is associated with stimulation condition. **b** The top contributing genes to Factor1 and their expression across cell types that are present across all samples. **c–d** MOFAtalk applied to inferred ligand-receptor interactions using LIANA. **c** The four factors inferred by MOFAtalk, where Factor 1 is significantly associated with biological condition. **d** Enriched ligand-receptor interactions for Factor 1.

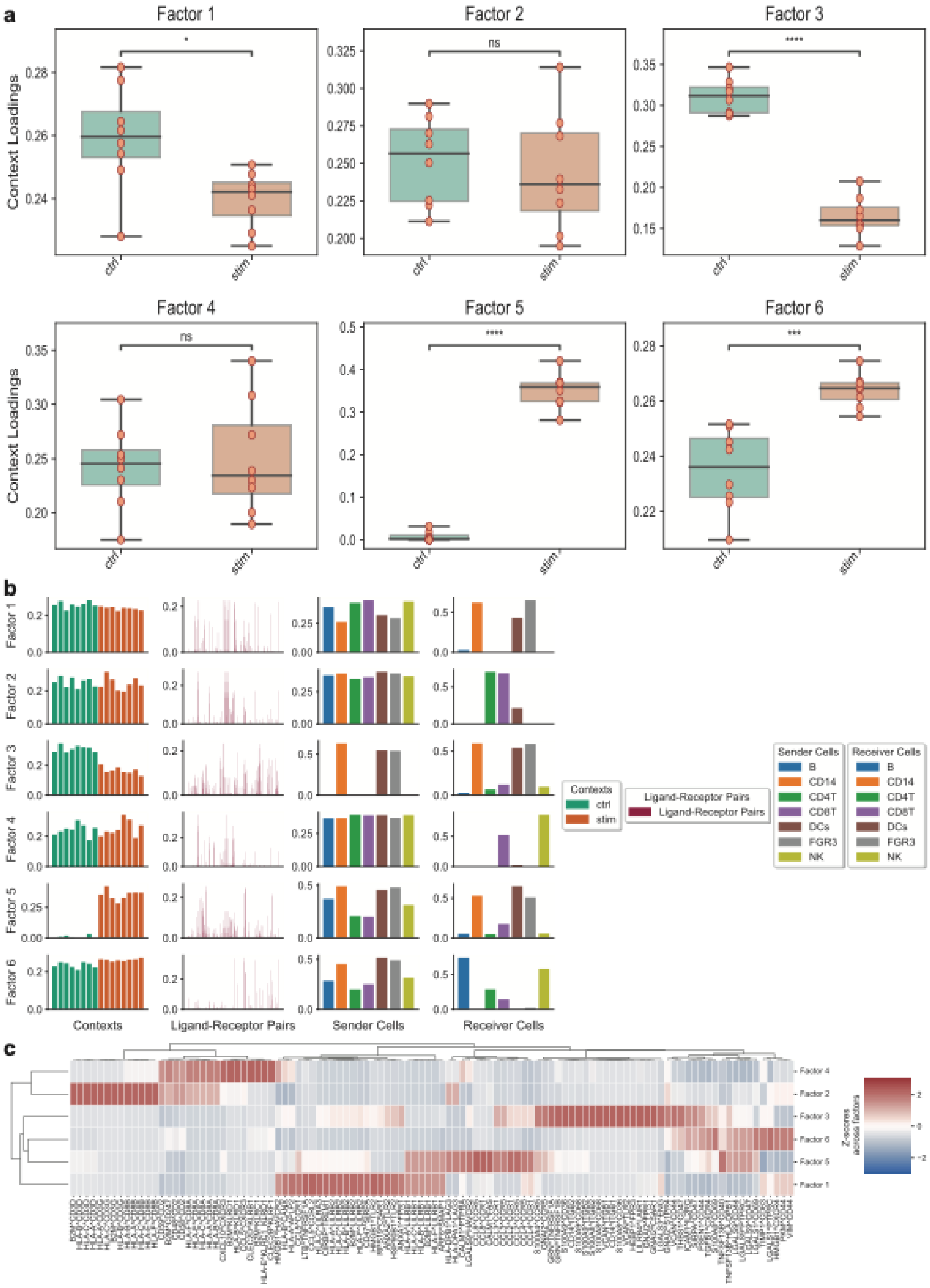

Supplementary Figure 5: Tensor-cell2cell applied to the Kang *et al.*[13] PBMC dataset. a Six factors were identified (eight replicates for ctrl, eight replicates for stim). Box plot whisker bounds are defined by minimum (ctrl: 0.25, 0.21, 0.29, 0.18, 3.43×10^{-11}, 0.21; stim: 0.22, 0.19, 0.13, 0.19, 0.28, 0.25) and maximum (ctrl: 0.28; 0.29, 0.33, 0.3, 0.03, 0.25; stim: 0.25, 0.31, 0.21, 0.34, 0.42, 0.27), while horizontal lines are defined by Q1 (ctrl: 0.25, 0.22, 0.29, 0.23, 10^{-6}, 0.23; stim: 0.23, 0.22, 0.15, 0.22, 0.32, 0.26), median (ctrl: 0.26, 0.26, 0.31, 0.25, 2.62×10^{-3}, 0.36, 0.24; stim: 0.24, 0.24, 0.16, 0.23, 0.36, 0.26), and Q3 (ctrl: 0.27, 0.27, 0.32, 0.26, 0.01, 0.25; stim: 0.25, 0.27, 0.18, 0.28, 0.37, 0.27). Statistical significance was inferred by calculating a two-sided t-test and p-values were adjusted using the Benjamini-Hochberg procedure. Here, ‘ns’ means p.adj < 1; ‘*’ means p.adj < 0.05; ‘**’ means p.adj < 0 .01; ‘***’ means p.adj < 10^{-3}; and ‘****’ < p.adj < 10^{-4}. Factor 1 (p.adj = 0.011), Factor 3 (p.adj = 3.722×10^{-9}), Factor 5 (p.adj = 2.196×10^{-12}), and Factor 6 (p.adj = 1.712×10^{-4}), are significantly associated with stimulation, while Factor 2 (p.adj = 0.677) and Factor 4 (p.adj = 0.731), were deemed not significant. **b** Factor loadings per sample and their enrichment across condition, ligand-receptor pairs, sender cells, and receiver cells. **c** Clustering of ligand-receptor interactions.

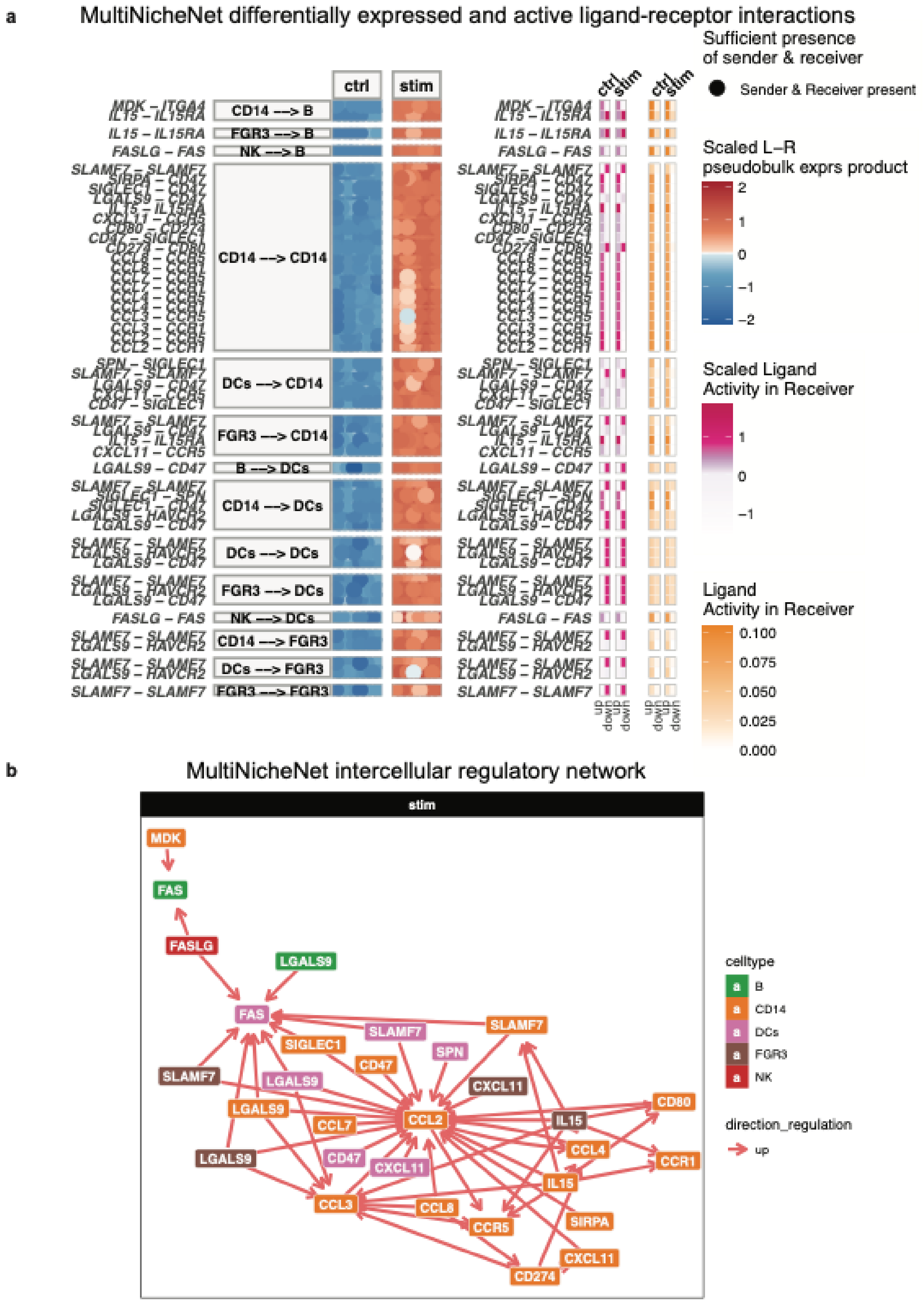

Supplementary Figure 6: MultiNicheNet applied to the Kang *et al.*[13] PBMC dataset. a Differentially expressed ligand-receptor interactions between condition inferred by MultiNicheNet. **b** The intercellular flow network inferred by MultiNicheNet.

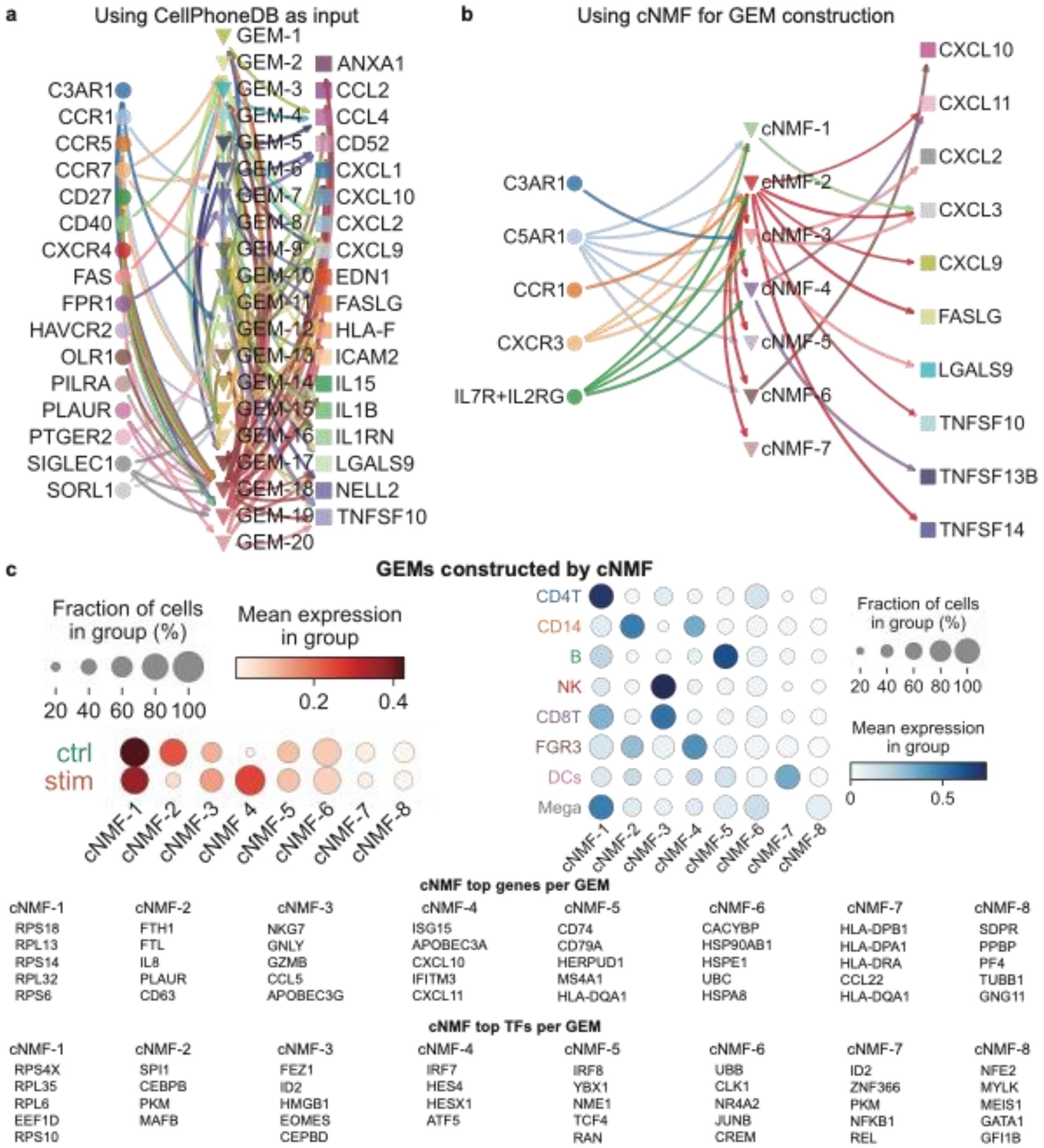

Supplementary Figure 7: FlowSig applied to the Kang *et al.* PBMC dataset with alternative inputs. a The intercellular flow network inferred by FlowSig where signal inflow and signal outflow variables are inferred from input generated by CellPhoneDB rather than input generated by CellChat. **b** The intercellular flow network inferred when using cNMF for GEM construction rather than pyLIGER (CellChat was used as input for signal inflow and outflow variables). **c.** GEMs constructed by cNMF are more enriched for cell type than stimulation type. The top five genes and TFs per GEM are listed.

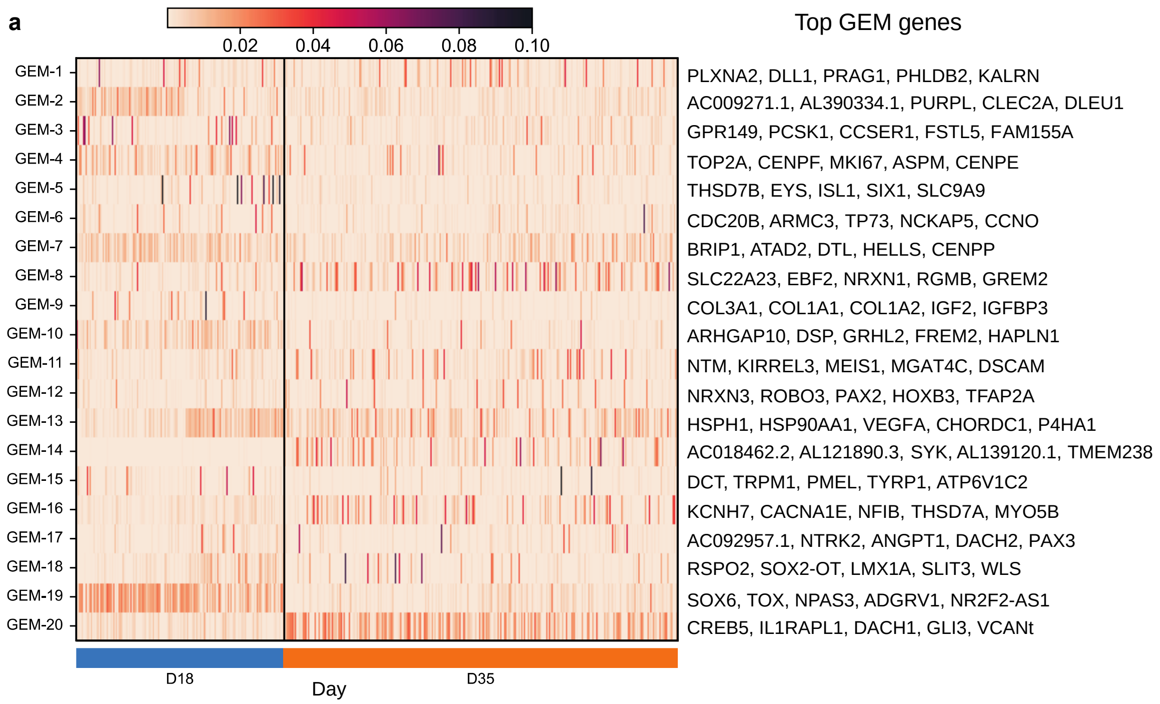

b Top GEM TFs

GEM-1	GEM-2	GEM-3	GEM-4	GEM-5	GEM-6	GEM-7	GEM-8	GEM-9	GEM-10
CBFA2T2	LIN28A	ONECUT3	HMGB2	ISL1	TP73	DTL	EBF2	ETS1	GRHL2
HES6	POU6F2	ZNF83		SIX1	EBF3	RFC3	NFIA	TWIST1	GLIS3
SETBP1	SND1	TBR1		EBF1	FOXJ1	MELK	NHLH1	BNC2	ESRP1
TFDP2	MDM2	POU2F2		POU4F1	EBF2	E2F7	KLF7	TFAP2B	IKZF2
HUNK		CELF		TBX3	EOMES	ZNF730	KLF12		
EBF2		PBX1		NEUROD1	NHLH1	E2F3	ZNF423		
VSX1		EBF1		EBF3	RORB	ZNF704	NHLH2		
NCOA1					LHX5		RFX8		
NEUROG1					E2F7				
SOX4									

GEM-11	GEM-12	GEM-13	GEM-14	GEM-15	GEM-16	GEM-17	GEM-18	GEM-19	GEM-20
MEIS1	PAX2	RBPJ		TFEC	NFIB	PAX3	LMX1A	SOX6	CREB5
PBX3	HOXB3	MXD1		MITF	ZEB2	PAX7	MECOM	GLI3	DACH1
FOXP2	TFAP2A	CHD2		RBMS1	NFIA	RFX4	NR3C2	ZNF536	GLI3
CELF4	ZFHX3	ATF3		ANXA1	HIVEP3	ZIC1	OTX2	GLI2	HMGA2
MEIS2	HOXA3				TBR1	HOXB3	POU6F2	NR2F1	NCALD
	RARB					HOXA3	CUX2	ID4	SOX5
	EBF1					MECOM		PAX6	FOXP1
	TFAP2B					HOXD3		ZNF521	ID4
	PAX8					NR3C1		ZNF462	PRDM16
	LUZP2					ZIC4		PDLIM5	TCF4

Supplementary Figure 8: Construction of gene expression modules from developing cortical organoid scRNA-seq. a Cell membership for each GEM and the top five GEM-specific genes, as ranked by GEM contribution weight, i.e. factor loadings. **b** Top TFs by contribution weight for each GEM.

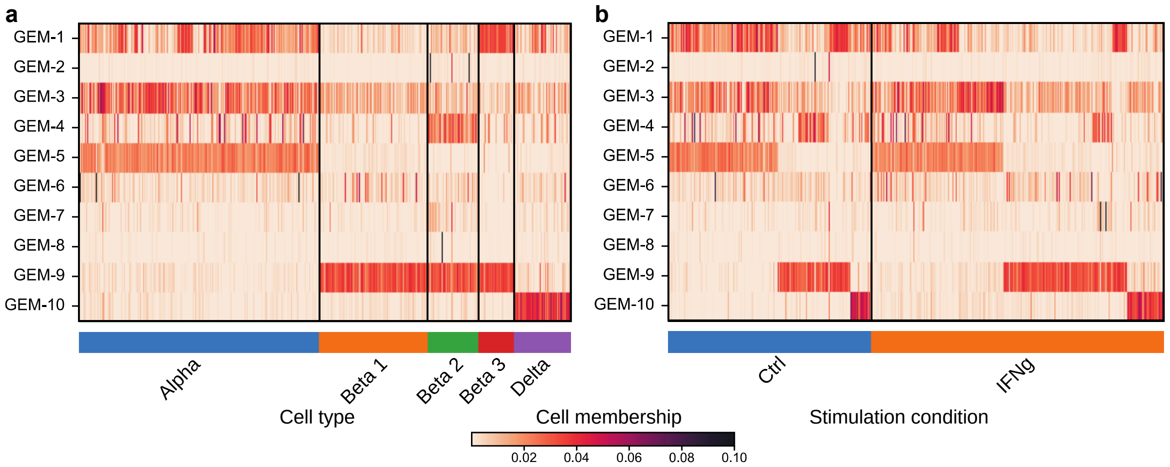

c

Top GEM genes

GEM-1	GEM-2	GEM-3	GEM-4	GEM-5	GEM-6	GEM-7	GEM-8	GEM-9	GEM-10
SERPIND1	NCOA7	SQLE	PTOV1	RPL36A	IGF2R	IFIT1	NBL1	CXCL6	NQO1
TSPYL1	BMP5	FBXW7	FABP4	RPL37	CALU	MUC6	GNS	NFIL3	HSPD1
LRP10	PGAM2	LAP3	LAMTOR5	RPL21	FDPS	ETV1	ARGLU1	SNX3	DDC
PPARGC1A	SORBS2	TXN	RP11-159D12.2	EPAS1	MEIS3	PSMB3	MAFF	CXCL17	HSD17B7
ITGB6	C5orf30	C1QB	TSPYL2	RPS27A	SP100	LAMTOR1	GOLGA4	CX3CL1	HSP90AB1

d

Top GEM TFs

GEM-1	GEM-2	GEM-3	GEM-4	GEM-5	GEM-6	GEM-7	GEM-8	GEM-9	GEM-10
PPARGC1A	PGAM2	PPP1R10	NKX6-1	EPAS1	MEIS3	ETV1	MAFF	NFIL3	GRHL2
CD59	OVOL1		TRIB1	SNAI2	SP100	IRX2	TPI1	YWHAZ	GLIS3
SCAND1	SIX3		ANXA11	RPS10	MEF2C	IRX3	REL	NFKB2	ESRP1
HIST2H2BE	IRF9,		ETV7	RPL6	RAD21	TAGLN2	ZBTB41	KLF3	IKZF2
PLAGL1	ENO1		RUFY3	RPL35	NPDC1	INSM1	SRF	FOS	
TCF7	EDN1			RPS4X	CTNNB1	NME1	ZNF467	NFE2L2	
EZR	KIF22			RPP25	JUNB	JUND	TAF7	ISL1	
MAFB	ELF3			MTF1	CD59	PSMD12	ZBTB21	EGR4	
SSBP3	DHX36YWHAE			EEF1D		EWSR1	HSF4	NFIC	
HES6				ZC3H7A		POU2F2		NFKB1	

Supplementary Figure 9: Construction of gene expression modules from stimulated human pancreatic islet scRNA-seq. a Cell membership for each GEM, where cells are sorted by cell type annotation. **b** Cell membership for each GEM, where cells are sorted by stimulation condition. **c** Top five genes per GEM as ranked by GEM contribution weight. **d** Top TFs per GEM as ranked by contribution weight.

a **Top GEM genes**

GEM-1	GEM-2	GEM-3	GEM-4	GEM-5	GEM-6	GEM-7	GEM-8	GEM-9	GEM-10
CBFA2T2<br>HES6<br>SETBP1<br>TFDP2<br>HUNK<br>EBF2<br>VSX1<br>NCOA1<br>NEUROG1<br>SOX4	LIN28A<br>POU6F2<br>SND1<br>MDM2	ONECUT3<br>ZNF83<br>TBR1<br>POU2F2<br>CELF<br>PBX1<br>EBF1	HMGB2	ISL1<br>SIX1<br>EBF1<br>POU4F1<br>TBX3<br>NEUROD1<br>EBF3	TP73<br>EBF3<br>FOXJ1<br>EBF2<br>EOMES<br>NHLH1<br>RORB<br>LHX5<br>E2F7	DTL<br>RFC3<br>MELK<br>E2F7<br>ZNF730<br>E2F3<br>ZNF704	EBF2<br>NFIA<br>NHLH1<br>KLF7<br>KLF12<br>ZNF423<br>NHLH2<br>RFX8	ETS1<br>TWIST1<br>BNC2<br>TFAP2B	GRHL2<br>GLIS3<br>ESRP1<br>IKZF2

GEM-11	GEM-12	GEM-13	GEM-14	GEM-15	GEM-16	GEM-17	GEM-18	GEM-19	GEM-20
MEIS1<br>PBX3<br>FOXP2<br>CELF4<br>MEIS2	PAX2<br>HOXB3<br>TFAP2A<br>ZFHX3<br>HOXA3<br>RARB<br>EBF1<br>TFAP2B<br>PAX8<br>LUZP2	RBPJ<br>MXD1<br>CHD2<br>ATF3		TFEC<br>MITF<br>RBMS1<br>ANXA1	NFIB<br>ZEB2<br>NFIA<br>HIVEP3<br>TBR1	PAX3<br>PAX7<br>RFX4<br>ZIC1<br>HOXB3<br>HOXA3<br>MECOM<br>HOXD3<br>NR3C1<br>ZIC4	LMX1A<br>MECOM<br>NR3C2<br>OTX2<br>POU6F2<br>CUX2	SOX6<br>GLI3<br>ZNF536<br>GLI2<br>NR2F1<br>ID4<br>PAX6<br>ZNF521<br>ZNF462<br>PDLIM5	CREB5<br>DACH1<br>GLI3<br>HMGA2<br>NCALD<br>SOX5<br>FOXP1<br>ID4<br>PRDM16<br>TCF4

b **Top GEM TFs**

GEM-1	GEM-2	GEM-3	GEM-4	GEM-5	GEM-6	GEM-7	GEM-8	GEM-9	GEM-10
	POU2AF1<br>MYBL2<br>XBP1	GATA2<br>HES4<br>MXD1<br>CREM<br>ZNF331	PRDX5<br>FOXJ1	CREB3L1<br>TFF3<br>SPDEF	HES1<br>ELF3	UBB<br>NR4A1<br>HSPA1L	ATF5<br>MAFB	ZNF683<br>RUNX3<br>IKZF3	HMGB2

GEM-11	GEM-12	GEM-13	GEM-14	GEM-15	GEM-16	GEM-17	GEM-18	GEM-19	GEM-20
BATF	ATF3<br>EGR2<br>CEBPB	ETS1<br>KLF2<br>RORA<br>TCF7<br>BCL11B	PITX1	NR4A3<br>TCF4<br>MEF2C<br>REL<br>BCL11A<br>SPIB	FHL2	TCF7L2<br>ZEB2		RPS4X<br>RPL6	NR1H3<br>DAB2

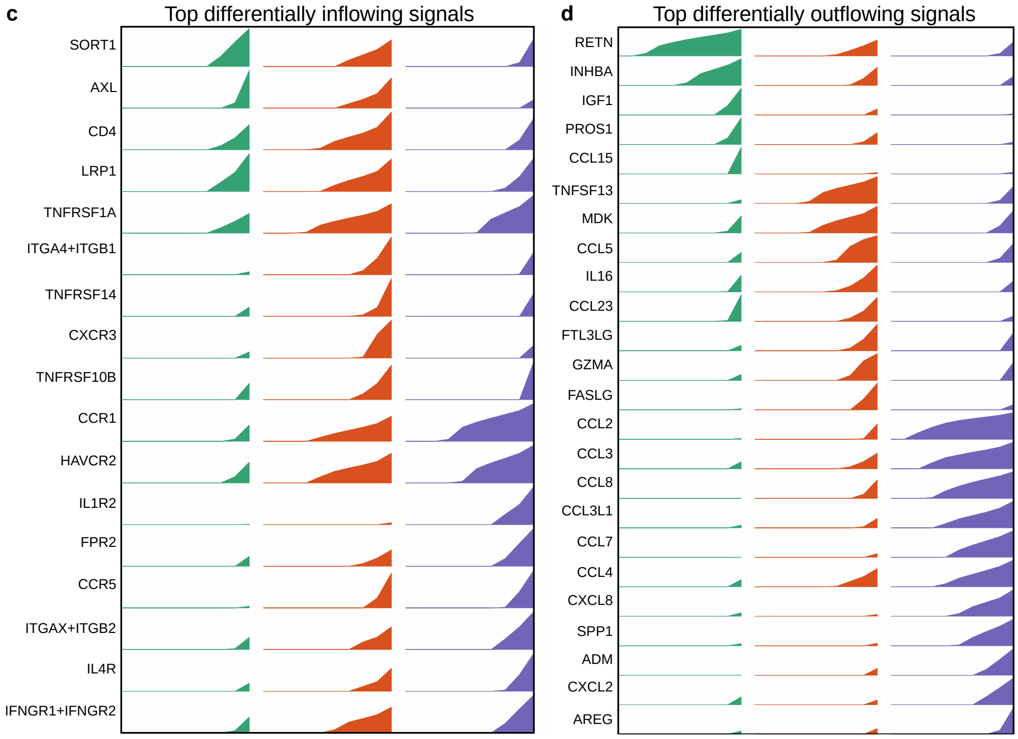

Supplementary Figure 10: Construction of gene expression modules and differential signal analysis from multi-condition human COVID-19 BALF scRNA-seq. a Top five genes per GEM as ranked by GEM contribution weight. **b** Top TFs per GEM as ranked by contribution weight. **c** Tracks plot of the top differentially inflowing signal variables per condition, where gene expression has been binned, and sorted by average expression per bin. **d** Tracks plot of the top differentially outflowing signal variables per condition, where gene expression has been binned, and sorted by average expression per bin.

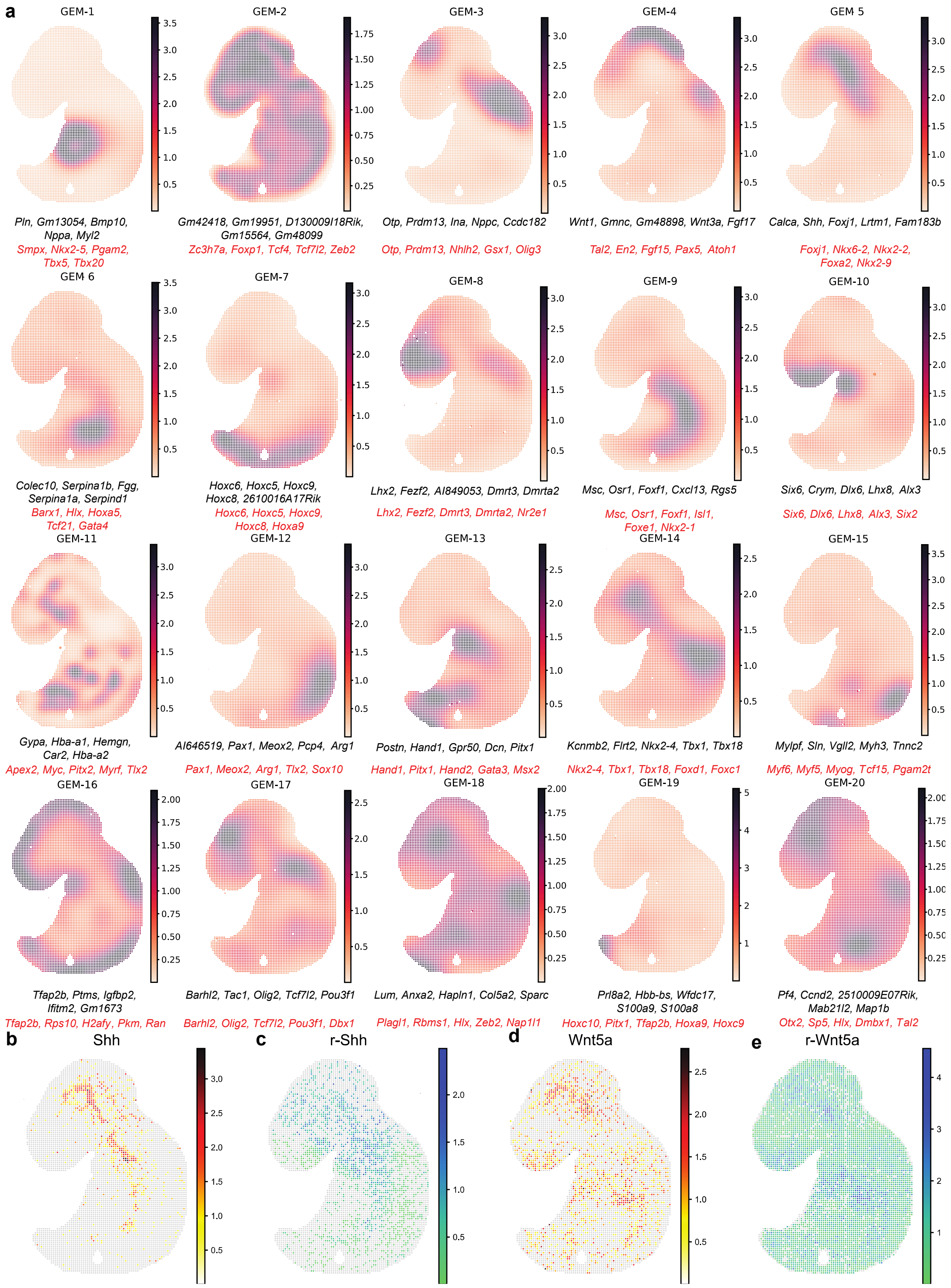

Supplementary Figure 11: Construction of gene expression modules from E9.5 mouse embryo spatial Stereo-seq. a GEM expression across spatial coordinates. The top five genes (black) and TFs (red) for each GEM are listed. **b** Spatial expression of outflowing *Shh.* **c** Spatial expression of inflowing *r-Shh* as inferred by COMMOT. **d** Spatial expression of outflowing *Wnt5a.* **e** Spatial expression of inflowing *r-Wnt5a* as inferred by COMMOT.
