# Supplementary material for: Inferring pattern-driving intercellular flows from single-cell and spatial transcriptomics
Source: Nat Methods. 2024 Aug 26;21(10):1806–17. doi: 10.1038/s41592-024-02380-w (PMC11466815; doi:10.1038/s41592-024-02380-w)
Supplement: Supplementary file 2 — Reporting Summary [file 41592_2024_2380_MOESM2_ESM.pdf]

nature portfolio

Corresponding author(s): Qing Nie

Last updated by author(s): Jun 10, 2024

Reporting Summary

Nature Portfolio wishes to improve the reproducibility of the work that we publish. This form provides structure for consistency and transparency in reporting. For further information on Nature Portfolio policies, see our Editorial Policies and the Editorial Policy Checklist.

Statistics

For all statistical analyses, confirm that the following items are present in the figure legend, table legend, main text, or Methods section.

n/a	Confirmed	
☐	☒	The exact sample size (*n*) for each experimental group/condition, given as a discrete number and unit of measurement
☒	☐	A statement on whether measurements were taken from distinct samples or whether the same sample was measured repeatedly
☐	☒	The statistical test(s) used AND whether they are one- or two-sided<br>*Only common tests should be described solely by name; describe more complex techniques in the Methods section.*
☒	☐	A description of all covariates tested
☒	☐	A description of any assumptions or corrections, such as tests of normality and adjustment for multiple comparisons
☐	☒	A full description of the statistical parameters including central tendency (e.g. means) or other basic estimates (e.g. regression coefficient) AND variation (e.g. standard deviation) or associated estimates of uncertainty (e.g. confidence intervals)
☐	☒	For null hypothesis testing, the test statistic (e.g. *F*, *t*, *r*) with confidence intervals, effect sizes, degrees of freedom and *P* value noted<br>*Give P values as exact values whenever suitable.*
☒	☐	For Bayesian analysis, information on the choice of priors and Markov chain Monte Carlo settings
☒	☐	For hierarchical and complex designs, identification of the appropriate level for tests and full reporting of outcomes
☐	☒	Estimates of effect sizes (e.g. Cohen's *d*, Pearson's *r*), indicating how they were calculated

Our web collection on statistics for biologists contains articles on many of the points above.

Software and code

Policy information about availability of computer code

Data collection	No software was used.
Data analysis	The code for the FlowSig is available on GitHub as a Python package (https://github.com/aalmet/FlowSig), along with all corresponding Python and R analysis code to reproduce the results (https://github.com/aalmet/FlowSigAnalysis_2023). The processed versions of the analyzed datasets are available at the following Zenodo repository: https://zenodo.org/doi/10.5281/zenodo.10850397<br><br>The following programming languages and versions were used:<br>Python 3.8.6<br>R 4.2.3<br><br>The following software packages and versions were used:<br>AnnData 0.9.2<br>causaldag 0.1a163<br>CellChat 1.6.1<br>CellPhoneDB 5.0.0<br>COMMOT 0.0.3<br>DIALOGUE 1.0<br>conditional_independence 0.1a6<br>graphical-model-learning 0.1a8<br>graphical-models 0.1a19<br>GraphPad Prism 9

joblib 1.3.1
liana 1.0.4
Matplotlib 3.7.2
Mofapy2 0.7.0
Mofax 0.3.6
multinichenetr 1.0.3
Networkx 3.1
NSF 0.0.1
Numpy 1.24.4
Omnipath 1.0.8
Pandas 2.0.3
pygam 0.8.0
pyLIGER 0.2.0
py-pde 0.36.0
PyTorch 2.0.1
Scanpy 1.9.3
Scipy 1.10.1
scITD 1.0.4
Seaborn 0.12.2
Squidpy 1.2.3

For manuscripts utilizing custom algorithms or software that are central to the research but not yet described in published literature, software must be made available to editors and reviewers. We strongly encourage code deposition in a community repository (e.g. GitHub). See the Nature Portfolio guidelines for submitting code & software for further information.

Data

Policy information about availability of data

All manuscripts must include a data availability statement. This statement should provide the following information, where applicable:

- Accession codes, unique identifiers, or web links for publicly available datasets
- A description of any restrictions on data availability
- For clinical datasets or third party data, please ensure that the statement adheres to our policy

The human cortical organoid scRNA-seq is available at NCBI GEO at accession number GSE239542 and will be released upon publication. Gene expression count matrices were constructed by aligning FASTQ files using Split-pipe v0.9.6 by Parse Biosciences. The alignment was done using the reference genome GRCh38.
The human pancreatic islet scRNA-seq data was originally published by Burkhardt et al.38; the raw gene expression counts and treatment condition metadata were downloaded from NCBI GEO at accession GSE161465. The scRNA-seq data of human COVID-19 patient BALF samples was originally published in Liao et al.40; the gene expression matrices and cell type annotation metadata were downloaded from NCBI GEO at GSE145926. The spatial Stereo-seq of mouse embryogenesis at time E9.5 was published originally in Chen et al.41; the annotated spatial data was extracted from the file “Mouse_embryo_all_stage.h5ad” hosted at https://db.cngb.org/stomics/mosta/download/.

Human research participants

Policy information about studies involving human research participants and Sex and Gender in Research.

Reporting on sex and gender	We used public data in the manuscript and human embryonic stem cell lines and there is no human research involved.
Population characteristics	We used public data in the manuscript and human embryonic stem cell lines and there is no human research involved.
Recruitment	We used public data in the manuscript and human embryonic stem cell lines and there is no human research involved.
Ethics oversight	We used public data in the manuscript and human embryonic stem cell lines and there is no human research involved.

Note that full information on the approval of the study protocol must also be provided in the manuscript.

Field-specific reporting

Please select the one below that is the best fit for your research. If you are not sure, read the appropriate sections before making your selection.

☒ Life sciences ☐ Behavioural & social sciences ☐ Ecological, evolutionary & environmental sciences

For a reference copy of the document with all sections, see nature.com/documents/nr-reporting-summary-flat.pdf

Life sciences study design

All studies must disclose on these points even when the disclosure is negative.

Sample size	No statistical sample size calculation was performed for this study. Instead, the sample sizes were determined based on practical

Sample size	considerations and the need to obtain a sufficient number of cells for subsequent analyses. For sequencing: at day 18 and day 35, two sets of samples were collected. We pooled 160 cortical organoids into one sample for D18, and we pooled 25 cortical organoids into one sample for D35. These numbers was chosen to ensure that we could obtain more than 1 million cells per sample, which was necessary to achieve sufficient material for the fixing and freezing steps prior to sequencing. For RT-qPCR: samples were pool from 4 organoids per sample and 2-4 samples per group. The rationale for these sample sizes is that RT-qPCR is a highly sensitive assay capable of detecting changes in gene expression even with relatively small sample sizes. Our results confirmed that pooling organoids in this manner provided enough RNA to detect meaningful differences in gene expression.
Data exclusions	Cells expressing fewer than 500 unique genes, or more than 10,000 genes were removed. Cells with more than 5% of their total gene expression contributed by mitochondrial genes were removed. No data exclusion for RT-qPCR experiment.
Replication	All experiments were at least in duplicates. All attempts at replication were successful and included in data analyses.
Randomization	Randomization is not relevant to this study. For sequencing: the samples had to be collected at specific time points and processed into single cell suspensions immediately. After all single cell suspensions were gathered, these samples were sent to sequencing core all together. For RT-qPCR: the control and experimental groups were from the organoids produced in the same batch. Sample preparations for control and experimental groups were collected at day 35 and RNA extractions were performed together.
Blinding	Blinding is not necessary for this study. For sequencing: the samples had to be collected at specific time points and processed into single cell suspensions immediately. Afterwards, the samples were handled by the core facility staff and the sequencing data handled by researchers who had no prior assumption of the biology, as this is an exploratory study. For RT-qPCR: RNA extractions were done for all samples at the same time in no particular order. Blinding is not relevant to the execution of RT-qPCR and technical replicates were included to avoid bias. Data analysis was done in the same way for all groups with no data exclusion.

Reporting for specific materials, systems and methods

We require information from authors about some types of materials, experimental systems and methods used in many studies. Here, indicate whether each material, system or method listed is relevant to your study. If you are not sure if a list item applies to your research, read the appropriate section before selecting a response.

Materials & experimental systems

n/a	Involved in the study
☒	☐ Antibodies
☐	☒ Eukaryotic cell lines
☒	☐ Palaeontology and archaeology
☒	☐ Animals and other organisms
☒	☐ Clinical data
☒	☐ Dual use research of concern

Methods

n/a	Involved in the study
☒	☐ ChIP-seq
☒	☐ Flow cytometry
☒	☐ MRI-based neuroimaging

Eukaryotic cell lines

Policy information about cell lines and Sex and Gender in Research

Cell line source(s)	Cortical organoids were generated using H9 (WA09; WAe009-A; WiCell) human embryonic stem cell. H9 originated from a female.
Authentication	The cell line was purchased directly from the WiCell under the MTA, has been tested negative for viral and mycoplasma infections, and regularly checked for chromosomal abnormalities. The H9 is de-identified for distribution from the WiCell.
Mycoplasma contamination	The H9 cell line was tested negative for mycoplama contamination.
Commonly misidentified lines (See ICLAC register)	No commonly misidentified lines.
